# Do minimum wage laws affect those who are not covered? Evidence from agricultural and non-agricultural workers

**DOI:** 10.1371/journal.pone.0221935

**Published:** 2019-10-02

**Authors:** Maoyong Fan, Anita Alves Pena

**Affiliations:** 1 Department of Economics, Ball State University, Muncie, Indiana, United States of America; 2 Department of Economics, Colorado State University, Fort Collins, Colorado, United States of America; TED University, TURKEY

## Abstract

Some employers are not obligated to pay at least minimum wages to all employees. U.S. farm employers comprise one of these groups. Employees of large farms and H-2A workers (lawfully admitted, nonimmigrant workers performing temporary or seasonal agricultural work) are protected by minimum wage legislation, while some migrant workers (often those paid piece rates) are exempt. U.S. agriculture also is characterized by a large percentage of unauthorized workers who may or may not earn above minimum wage. Following insights from dual labor market theory and from theories of the signaling capacity of the minimum wage, we compare labor market outcomes in the agricultural sector (where minimum wage coverage is limited) to low wage/skill non-agricultural sectors (where minimum wage coverage is more complete) nationally using data from the Current Population Survey. We then extend our analysis to a detailed state-level case study of agricultural workers in California using a representative survey of employed farm workers. Results suggest wage increases for covered workers that exceed those for uncovered workers, but insignificant differences in hours worked. This is the first study to our knowledge to examine the impacts of minimum wage coverage on agricultural workers relative to other workers for the U.S.

## Introduction

Despite ongoing political discussion, the federal minimum wage has not increased since it became $7.25 in July 2009. Many states (and some localities) have their own minimum wage laws which increase state minimum wages much above the federal level. In the case of conflicts between applicable minimum wage levels, workers are entitled to the highest rate. Minimum wage coverage, however, is not universal. Some employers of low-income workers are not obligated to provide minimum wages to all employees. Exemptions to the federal minimum wage currently apply under specific circumstances to workers with disabilities, full-time students, those under age 20 in their first 90 consecutive calendar days of employment, tipped employees, student learners, workers in seasonal and recreational establishments, salaried executive, administrative, professional and outside sales employees, some home aides, and agricultural workers [1]. We study the impacts of minimum wage on agricultural workers relative to other low-wage workers in this research.

The Fair Labor Standards Act (FLSA) of 1938 established federal rules regarding minimum wages, overtime pay provisions, and child labor standards. Until amendments to the Act in 1966, agriculture was completely excluded. Agricultural and nonagricultural wages were in fact not deemed subject to common standards completely until additional revisions in 1978. Today, most agricultural employees should be covered by minimum wages statutorily since they produce goods for interstate commerce. However, several exemptions specific to this sector result in many workers remaining uncovered in practice. First, small farms that do not use 500 “man days” per year are exempt from paying minimum wages. Second, employers are not required to pay minimum wages to several groups of workers, including immediate family members, those principally engaged in livestock production, local piece rate paid hand harvest laborers (i.e. workers paid by specific productivity as opposed to by time worked), and nonlocal minors under 16 years of age in piece rate hand harvesting alongside his or her parents [1]. Furthermore, compliance with minimum wage legislation may be limited in agriculture, especially in the case of unauthorized immigrant workers.

Although much academic literature has focused on the employment effects of minimum wage increases on workers for whom these wages directly apply ([2–8] and others), these results are controversial regarding methodologies and data used and results found. Our focus in contrast to this past work is on the less-studied effects of minimum wage legislation, particularly those effects on wages and hours worked of workers who are not explicitly covered. Theoretically, we may expect that wages of both covered and non-covered workers will increase in response to minimum wage legislation given that the outside option value (opportunity cost) for non-covered worker has increased. This is consistent with interpretations of the “lighthouse effect,” a notion in the literature of how bargaining power may change for all workers following minimum wage increases due to signaling by these increases regarding labor market conditions [9].

Sorting of workers and labor market tightening following minimum wage changes in uncovered relative to covered sectors can create further upward pressures on uncovered wages. These impacts, however, could be offset if demand for workers in the higher-paid covered sector decreases enough to push substantial numbers of workers back into the uncovered sector. In addition, hours worked theoretically may decrease or increase depending on whether researchers model the labor market as perfectly competitive or as monopsonistic and depending on whether effects are envisioned to operate on individual hours (intensive margin) or on total employment itself (extensive margin). Impacts of minimum wage changes on uncovered workers therefore become theoretically ambiguous, motivating the importance of empirical investigation.

Literature on minimum wages in the context of agricultural labor specifically is limited. Early papers found that the extension of some minimum wage coverage to agricultural workers had a positive effect on wages but at the expense of the size of the total agricultural labor force [10–12]. These papers primarily looked at the introduction of minimum wages over time as opposed to differences in minimum wages across time and space as we do here. In addition, little literature has examined minimum wage coverage in the context of agriculture in comparison to other sectors in the U.S., and we aim to contribute by filling this gap.

We use data from the Current Population Survey (CPS) to test whether the minimum wage impacts agricultural workers, who are less likely to be covered, in a way that is different from workers who are in other low skilled sectors. We supplement this cross-sector comparison with additional results for the case study of California from the National Agricultural Workers Survey (NAWS), which is a distinctive and lesser-used dataset that has the advantage of allowing analysis by legal status (i.e. unauthorized versus authorized) and by payment basis (i.e. piece rate versus time rate), both features of which are closely linked to minimum wage coverage and its enforcement.

Our results suggest that wage increases for covered workers exceed those for uncovered workers, and that there are insignificant differences across these groups in hours worked before and after minimum wage changes. Although these results overall match theoretical expectations given the non-bindingness of the minimum wages in the uncovered sector, this is the first study to our knowledge to examine the impacts of minimum wage coverage on agricultural workers relative to workers in plausible substitute employment sectors for the case of the U.S. Furthermore, our extensions to the examination of workers in piece rate versus hourly paid positions in agriculture and those who are undocumented versus documented represent further contributions and empirical confirmation of theoretical mechanisms operating differentially on subsets of the relevant worker population.

## Literature and theoretical considerations

Literature on minimum wages primarily has focused on employment and wage effects within the covered sector. According to mainstream economic models, increasing minimum wages should have adverse effects on total employment. Some empirical work, however, has failed to confirm this pattern. In seminar research, Card and Krueger (1994), for example, finds that minimum wages do not lead to decreases in employment for a case of the fast food industry [4]. This result has been debated in subsequent literature [5–6], and authors have argued that differences may be due to competition characteristics of establishments employing low-wage workers [2] or empirical judgments relating to the use of year effects as macroeconomic controls [3]. Sabia (2009) exploits variation from recent minimum wage changes and estimates negative labor demand effects on teenagers using CPS data and alternative macroeconomic controls (such as whether the economy is in recession and the proportion of the population that is teenaged) in contrast to earlier work [13]. Meer and West (2015) complement this work, finding that real employment impacts associated with minimum wages may operate through growth rates [14].

### Minimum wages and the uncovered sector

Arguably, examining general equilibrium effects of the minimum wage is important to further understanding the complementarity or substitutability of workers of various wage levels employed in covered versus uncovered sectors. We also argue that it is important to understand wage spillovers within and across sectors. Some papers have examined relationships between covered and uncovered workers in the presence of minimum wage legislation, though these papers start from differing theoretical perspectives and judgement calls regarding the appropriate status quo in terms of competition dynamics (e.g., [2–6]). Monopsonistic labor market models would suggest that uncovered workers, in addition to covered workers, may receive a wage boost in response to minimum wage increases since the existence of a more generous outside option may put upward pressure on uncovered sector wage rates if workers are free to move between sectors. In the monopsonistic model presented by Bhaskar and To (1999), an increase in the minimum wage may lead to an increase or a decrease in employment with resulting positive and negative welfare effects respectively [2].

Perfectly competitive models of the labor market yield predictions of increased wages but decreased employment in the covered sector. Effects on wages in a secondary uncovered market are ambiguous and dependent on extent of substitutability of workers, elasticities, and the magnitudes of labor demand and supply shifts. Minimum wages also affect opportunity costs of working in industries such as agriculture by influencing pay and employment probabilities in nonexempt jobs. Minimum wage increases might force workers from the nonexempt sector to the lower wage exempt sector, and this migration may lead to lower wages in the uncovered sector. These dynamics are sometimes referred to as negative spillover effects to the uncovered sector following minimum wage increases.

Worker sorting between the uncovered and covered sectors may be systematically related to worker characteristics. We therefore expect the elasticity of labor supply to differ before and after minimum wage changes. This has the implication that the composition of “types” of workers available to employers in each of these sectors may vary substantially over time, as may the tightness of the labor market. Standard theoretical modeling relating labor market tightness to minimum wage dynamics predicts that as labor market tightness increases so does the return on search effort [15–16]. Minimum wage increases then should induce behavioral increases in search effort for those workers who are mobile across the uncovered and covered sectors.

Mincer (1976), in his early depiction of a dual labor market, writes that “minimum wages generate socially wasteful labor mobility” (p. S87) [17]. Specifically, he argues that minimum wages lead to both inefficient allocations of people between in and out of the labor force, and across covered and uncovered sectors within the market. His empirical analysis suggests that primary effects are the result of labor moving from covered to uncovered sectors after minimum wage increases, which puts downward pressure on uncovered wages through an increased labor supply that more than offsets changing option values. Tauchen (1981) further examines effects of minimum wages across covered and uncovered U.S. labor market sectors [18]. He estimates reduced-form wage and employment equations using aggregate data comparing the generally uncovered agricultural sector (prior to the FLSA) to four low-wage, covered nondurable manufacturing industries, and finds that uncovered sector wage rates increase following minimum wage increases in regions characterized by small covered-sector demand elasticities. Furthermore, uncovered wage rates fall in regions with large covered demand elasticities. This is consistent with Mincer’s (1976) minimum-wage model and indicates that context may matter for the determination of the incidence of the effects of minimum wage increases [17].

A mechanism referred to as a “lighthouse effect” in more recent literature purports that minimum wage increases in the formal sector may spillover to the informal sector via the signaling power of the minimum wage and corresponding changes to bargaining power of all workers [19]. The lighthouse effect framework suggests that minimum wage changes should increase the average wage in what has been called the “uncovered,” “informal,” or “shadow” sector in the literature. Boeri, Garibaldi, and Ribeiro (2011) extend this argument by showing how sorting across formal and informal sectors through an employer-employee matching process can explain some but not all the increases in wages in informal settings [9]. Their model predicts that the minimum wage changes the skill composition in the informal sector in the increasing direction since some low productivity workers exit to join the formal sector. The lighthouse effect modeling suggests that there are two types of workers in the post-minimum wage equilibrium in the formal sector. Some of these workers are of the type that would have been in the formal sector regardless and others are lower productivity movers into this sector (who effectively come from the lower part of the skill distribution within the original informal sector). Boeri, Garibaldi, and Ribeiro (2011) extend their analysis to an application using Brazilian data which they find to be consistent with this conceptual framework [9].

### Minimum wage and U.S. agricultural labor markets

Early agricultural labor economics literature considered the effects of the FLSA and its amendments on equilibrium outcomes. Most of this work has implicitly treated the agricultural and nonagricultural labor markets as being stratified, consistent with theories of dual labor markets [20–21].

Gardner (1972) and Lianos (1972) find that initial extensions of minimum wages to agricultural workers under the FLSA led to significantly increased wages and decreased employment [10–11]. Specifically, Gardner (1972) regresses a farm wage index on prices of land and other inputs, manufacturing wages, product prices, a time trend, and minimum wage introductions [10]. He finds that minimum wages that are applicable to agricultural labor markets are positively related to farm wages whereas the opposite is true for minimum wages that do not directly apply. Hired agricultural labor force sizes, on the other hand, are increasing in non-applicable minimum wages and decreasing in applicable minimum wages. Lianos (1972), in independent research, comes to similar conclusions and stresses that welfare effects of minimum wages on hired farm workers are characterized by winners and losers with some workers remaining employed at higher wages and others losing employment altogether [11]. These results are qualitatively similar to findings pertaining to the introduction of minimum wages in English and Welsh agriculture in 1917 (repealed in 1921 and reinstated in 1924). Gowers and Hatton (1997), for example, find that wage rates within agriculture increased substantially, while employment fell with reduced poverty to workers retaining their jobs [22].

Subsequent work also finds increases in wages and decreases in employment in the farm sector [22–24]. Gilroy (1982) confirms this basic result using proprietary federal data on coverage [24]. Mishra and Rezitis (1998) reproduce and update an equation like that in Gardner (1972) and document negative labor supply elasticities in agriculture [10, 12].

In more recent agricultural labor economics literature, Moretti and Perloff (2000) find that increases in the minimum wage raised wages for hourly workers in U.S. agriculture and decreased wages for piece rate workers on average during the 1989 to 1995 time period [25]. The authors present a general equilibrium model illustrating how wage effects are uncertain under an assumption of incomplete enforcement (incomplete coverage) of the minimum wage. Finally, Buccola, Li, and Reimer (2011) find that the labor supply elasticity in Oregon agriculture is high and that minimum wage changes have compressed the wage distribution [26]. The authors find that a dollar increase in minimum wages is associated with only about $0.31 in increased wages on average.

In a recent working paper, Hill (2018) shows theoretically and in an empirical application to California strawberry picking that an increase in minimum wages is associated with decreases in effort and productivity for piece rate paid workers [27]. This suggests that minimum wage effects could be different depending on payment type (e.g., hourly wage versus piece rate pay).

Some recent papers have examined uncovered workers in sectors other than our chosen sector of agriculture. For example, Dube, Lester, and Reich (2010) and Addison, Blackburn, and Cotti (2012) examine tipped restaurant workers [8, 28]. These workers may be different given the availability of higher wages in the presence of good tipping. However, features of their employment may share similarities with piece rate workers in agriculture which we study in sub-sections of this paper in more detail.

## Empirical framework

Theory suggests significant positive effects of minimum wages on wages received, even in the uncovered sector, as the labor market there becomes tighter as the size and composition of the covered and uncovered workforces change. On the other hand, labor demand is expected to decrease in the covered sector with minimum wage increases while individual search effort to match with a high-paying job increases. If this causes enough workers to return to the uncovered sector, the final equilibrium wage in the uncovered sector could be higher or lower. Predictions also can be ambiguous if regulations are complex and if employers are uncertain regarding the applicability of minimum wage laws to their workers. These dynamics support the need for the empirical study which we present here.

We expect differences across piece rate and time rate workers with piece rate workers having less of a response (due to labor law exclusions for this group). Similarly, we expect differences across undocumented and documented worker groups. Finally, we expect minimum wage effects on wages received to be highest for workers with observed wages closest to minimum wage levels.

Given the theoretical ambiguities, we build several empirical tests in order to learn more about the impacts of minimum wages across workers with different coverage. After a comparison across sectors, we examine wages within the agricultural sector where coverage is incomplete using more specialized data to isolate specific differences.

### Minimum wages and empirical techniques

The appropriateness of empirical strategies toward identifying the effects of minimum wage increases has been a topic of academic controversy, and this has culminated in the recent empirical literature on minimum wage effects within the field of economics. Neumark and Washer (2011) and Neumark, Salas, and Wascher (2014) critique common approaches such as the use of linear time trends for controlling for spatial heterogeneity in employment equations when estimating the effect of minimum wages [29–30]. They argue that time period may still matter in these specifications especially in the presence of more economically significant recessions in applications presented by other authors [31]. Following this debate, Allegretto et al. (2017) refute the updated techniques in Neumark, Salas, and Wascher (2014) [30, 32]. Their primary follow-up critique presented in this rebuttal relates to pre-existing differences in trends at the state-level. We include these insights in our empirical approach that involves nonlinear state-time trends.

### Relationships between minimum wages and wages received across sectors with different coverage

Because we are interested in the differential impacts of minimum wages on wages received across sectors with differential coverage, our empirical strategy focuses on a specification with a key interaction parameter of interest. We start by modeling a log wage equation where wages per hour received by person *i* are a function of minimum wages in state *s* and year *t*, a function of sector of employment, and a function of the interaction between minimum wage level and the employment sector where the employment sector is defined by agriculture versus other employment. Building upon canonical modeling of minimum wage impacts and its further development over time [32–33], we estimate a model with census division-period fixed effects and state-specific time trends. The model specification takes the form:

$$Y_{\mathrm{ist}}=\alpha \ln\left(\min\_w_{\mathrm{st}}\right)+{\beta \mathrm{agriculture}}_{\mathrm{ist}}+\gamma \ln\left(\min\_w_{\mathrm{st}}\right)\times {\mathrm{agriculture}}_{\mathrm{ist}}+X_{\mathrm{ist}}\theta +\pi_{s}+\delta_{\mathrm{dt}}+\sum_{k}\left(\varphi_{\mathrm{sk}}\times t^{k}\right)+\epsilon_{\mathrm{ist}}$$ (1)

where min_w_st_ is defined as the legislated minimum wage level in the state and time period (year) that the worker is observed (constructed as the higher of the applicable federal or state minimum wage). Sector of employment is represented by a dummy variable agriculture_ist_ which takes a value of one when the sample respondent works in the agricultural sector and zero when the respondent works in one of the sectors appearing in the base group. This inclusion, representing an extension of the canonical model, along with the interaction term between minimum wage level and agricultural employment, allows us to examine differences in the impact of minimum wage across a sector in which coverage is incomplete (agriculture) versus the control sectors. The dependent variable *Y*_*ist*_ is either the log of hourly earnings or the number of weekly hours. We then include a variety of control variables, *X*_*ist*_, including age, sex, minority race (as captured by a black indicator and a Hispanic indicator), categorical education level variables, a dummy variable for marital status, and indicators of firm size. The most saturated model includes up to fifth-order state-specific time trends and allow the time effects to vary by each of the nine census regions to control for the business cycle in addition to other variations and trends by year and across space. All specifications include state fixed effects (π_s_), and Census division-year fixed effects (δ_dt_). We cluster standard errors at the state level.

Examining weekly hours worked as an outcome variable, in addition to wages, allows us to draw further conclusions about the intensive margin of possible employment impacts. We also relax assumptions about the relevant part of the wage distribution in extensions. Orrenius and Zavodny (2008) find that more than 90 percent of low-skill native, immigrant, and teenage workers are making less than 300 percent of the minimum wage [34]. While in our main analysis, we consider everyone earning less than 300 percent of minimum wage in a nationally representative sample in order to focus on the group for which the minimum wage is closest to binding, we relax this assumption by changing the sample to those within 200 percent and within 250 percent of the minimum wage respectively in extensions.

Given controversy in the empirical literature of minimum wages over time, as indicated the previous discussion in this paper, we add specifications with increased attention to parallel trends and particularly the geographic clustering of high minimum wage states and economic clustering in terms of business cycle trends). These specifications account for time-varying, state-level heterogeneities.

### Relationships between minimum wages and wages received within agriculture

The agricultural sector is distinctive in that some workers are covered by minimum wage legislation while others are not. While the first specifications examine limited coverage versus coverage by looking at the agricultural sector in comparison to other low wage workers, specific individual workers who are covered or not cannot be identified precisely by either the data used or by the empirical approach taken. We extend our analysis by using separate data on agricultural workers and their individual characteristics in order to examine impacts of coverage on this sub-population more directly.

We examine workers who are paid by piece rate and by time rate separately in addition to our full sample as these workers have differential minimum wage coverage by current labor laws for a case study corresponding to California agriculture [35]. Related to this is whether being paid piece rate is the result of a choice on the part of the worker, or alternately is the result of a type of discrimination. Evidence on the prevalence and characteristics of farm labor contracting suggests that piece rate pay may not fully be a choice. Approximately 34 percent of piece rate workers, for example, report being employed by a farm labor contractor as opposed to directly by a grower, while only 15 percent of hourly workers report using farm labor contractors and wage gaps are evident between these two classes of workers. This suggests that individual workers may have more bargaining power when they represent themselves directly to a grower as opposed to via a third party.

We also examine differentials by legal status since unauthorized workers are not legally covered by minimum wages. These workers may or may not experience impacts like authorized workers.

Our primary specification in this second part takes the form:

$$Y_{\mathrm{it}}=\alpha \ln(\min\_w_{t})+{\beta \mathrm{unauthorized}}_{\mathrm{it}}+\gamma \ln(\min\_w_{t})\times {\mathrm{unauthorized}}_{\mathrm{it}}+X_{\mathrm{it}}\delta +\epsilon_{\mathrm{it}}$$ (2)

where *min*_*w*_*t*_ is the legislated minimum wage rate in California and unauthorized workers are compared via a dummy variable to all legal workers (U.S. natives, naturalized citizens, Green Card holders, and other work authorization) and a variety of other variables are controlled. The dependent variable *Y*_*it*_ is either the log of hourly earnings, a binary indicator for receiving a bonus, or the number of weekly hours. Control variables in this part are age, sex, education, marital status, farm work experience in years, tenure with current employer in years, English speaking ability, and a cubic time trend. These control variables differ from those for the estimation of Eq (1) due to the differing aims across the two parts of the analysis and different information available across the two surveys described below.

## Data and results

Our data come from three primary sources. First, we use state-specific minimum wage levels by year as reported by the U.S. Department of Labor. Second, we use nationally representative microdata from the Current Population Survey in order to examine broad patterns between the agricultural sector and other low-wage sectors in the U.S. Third, we introduce specialized data on agricultural workers specifically from the NAWS for the case of California (which is the only identifiable state in this survey) in order to more closely examine differences between workers who are paid piece rate versus hourly and between workers who are unauthorized and who are authorized. In both of our primary microdata sources, we use pooled cross-sectional samples for our empirical analysis.

### Current population survey

We use the March CPS data from 1990 through 2014 for the primary analysis of workers of low-skill occupations in the economy and to compare with the experience of agricultural workers. We elected to not use the Merged Outgoing Rotation Group (MORG) data from the CPS due to the migratory nature of hired agricultural workers and the related lower probability that workers would remain in the sample according to the MORG sample design. We chose these years to most closely overlap with the years of the specialized agricultural worker sample which we present in the second part of the empirical analysis. In March of each year, all workers in the basic CPS sample are administered a supplemental questionnaire in which they are asked to report income such as hourly wage rate and labor force activity such as hours worked last week.

Based on the 1990 occupation codes, we choose three other low-skill occupations as a comparison group for our analysis: construction workers, hotel service workers, and restaurant workers. Agricultural workers are those coded from 479 to 484 in the CPS variable occupation 1990. Construction workers are those coded from 559 to 599 and 869. Hotel workers are those coded as 453. Restaurant workers are those coded from 434 to 444. It is possible that some restaurant workers may be exempt via their roles as tipped servers. All workers who are 16 through 65 years old are included in the sample. We use appropriate sample weights (which vary by outcome variable) in the empirical analysis.

### Baseline models for wages and hours worked

We analyze summary statistics of our CPS data in Table 1. We see that there are many demographic and work-related differences between agricultural workers and those in construction, hotel and restaurants which we have chosen as comparison low-skill occupations. We present data for workers overall (across low and high-skilled occupations) and for workers in agriculture and in these other low-skill occupations respectively. To focus on the relevant part of the wage distribution, we restrict the overall sample to workers within 300 percent of their state’s minimum wage. We relax this assumption in supporting information as an extension.

**Table 1:** Summary statistics (current population survey).

	Full Sample	Agricultural Workers	Non-Agricultural Workers	Construction, Hotel, and Restaurant Workers
*Continuous Variables*				
Ln Hourly Earnings ($2016)	2.499	2.288	2.500	2.317
	(0.358)	(0.252)	(0.358)	(0.445)
Weekly Hours	34.63	34.84	34.63	32.24
	(11.39)	(13.35)	(11.38)	(11.87)
Age (Years)	36.11	33.86	36.12	33.61
	(12.94)	(13.44)	(12.94)	(13.13)
*Binary Variables*				
Female	0.559	0.199	0.561	0.409
Black	0.120	0.0338	0.120	0.105
Hispanic	0.132	0.406	0.130	0.186
Some School	0.213	0.542	0.211	0.339
High School Graduate	0.366	0.280	0.366	0.371
Some College	0.323	0.120	0.324	0.239
College and Above	0.0955	0.0286	0.0960	0.0447
Married	0.485	0.484	0.485	0.395
Employed Full Time	0.658	0.659	0.658	0.559
Firm Size (500–1000)	0.0590	0.0295	0.0592	0.0459
Firm Size (1000+)	0.381	0.0798	0.383	0.315
Number of Observations	161,729	1,153	160,576	27,253

Notes: The March CPS sample includes everyone with hourly salary less than 300% of state minimum wage. The sample period is from 1990 through 2014. The base group for education is no school. The base group for firm size is less than 500 people. According to 2012 Census of Agriculture, top 10 states with the most hired agricultural workers are California, Washington, Texas, Florida, Oregon, Michigan, Minnesota, Iowa, Wisconsin, and North Carolina.

The samples of agricultural workers are relatively small in comparison to the broader data on workers in other occupations. There are 1,153 valid observations of agricultural workers over this time period in comparison to more than 27,000 construction, hotel and restaurant workers represented in the CPS data. These are in comparison to more than 161,000 total observations in the full sample.

It is evident from the summary statistics in Table 1 that agricultural workers and workers in construction, hotel and restaurants are similar in terms of average age and in the distribution of age as indicated by the standard deviations in parentheses. These averages are notably lower than that for non-agricultural workers more broadly in the CPS. Agricultural workers are much more highly male and more likely to be Hispanic than are workers in our comparison sectors. Agricultural workers are weighted toward the lower part of the educational category distribution but are more likely to report being married and to be employed full time. A higher fraction of construction, hotel, and restaurant workers report working in very large firms (more than 1,000 employees) than do agricultural workers.

Agricultural workers have systematically lower wages but higher hours worked on average in comparison to the comparison occupations studied. Wages in both categories are lower than in the aggregate population as measured via the full sample statistics in Table 1. All wages are adjusted to 2016 real U.S. dollars using the Consumer Price Index.

As suggested by the conceptual discussion, overall patterns of the impacts of the minimum wage on agriculture may be different from those on workers in other low-skill occupations for reasons related to the degree of coverage of the minimum wage in these sectors. Our estimation results of Eq (1) are presented in Table 2. In these specifications, we find that the minimum wage is a statistically significant predictor of hourly wages across all specifications (columns (1) through (10)). Since we measure minimum wage and likewise hourly wages in natural log terms, we interpret coefficients on our minimum wage variable in terms of elasticities. For the full sample, a one percent increase in the minimum wage is associated with an approximately 0.4 percent increase in hourly earnings. For the subsample of low-skill workers (agriculture and comparison occupations), a one percent increase in minimum wage is associated with an approximately 0.5 to 0.6 percent increase in hourly earning all else equal.

**Table 2:** Minimum wage law and hourly wages.

	Full Sample					Low-Skill Sectors				
	(1)	(2)	(3)	(4)	(5)	(6)	(7)	(8)	(9)	(10)
Ln Minimum Wage	0.38**	0.39**	0.39**	0.40**	0.37**	0.50**	0.48**	0.51**	0.58**	0.51**
	(0.03)	(0.03)	(0.03)	(0.03)	(0.03)	(0.07)	(0.07)	(0.07)	(0.08)	(0.08)
Ln Minimum Wage × Agriculture	-0.40**	-0.40**	-0.40**	-0.40**	-0.40**	-0.57**	-0.58**	-0.58**	-0.58**	-0.59**
	(0.08)	(0.08)	(0.08)	(0.08)	(0.08)	(0.13)	(0.13)	(0.13)	(0.13)	(0.13)
Agriculture	0.64**	0.64**	0.65**	0.65**	0.65**	1.04**	1.05**	1.05**	1.06**	1.07**
	(0.17)	(0.17)	(0.17)	(0.17)	(0.17)	(0.26)	(0.26)	(0.26)	(0.26)	(0.26)
α + γ	-0.02	-0.01	-0.01	0.00	-0.03	-0.08	-0.10	-0.07	0.00	-0.08
	(0.09)	(0.09)	(0.09)	(0.09)	(0.09)	(0.15)	(0.15)	(0.15)	(0.14)	(0.14)
Number of Observations	161,729	161,729	161,729	161,729	161,729	28,406	28,406	28,406	28,406	28,406
R-squared	0.26	0.26	0.26	0.26	0.26	0.30	0.30	0.30	0.31	0.31
Controls	Yes	Yes	Yes	Yes	Yes	Yes	Yes	Yes	Yes	Yes
Division-Year FE	Yes	Yes	Yes	Yes	Yes	Yes	Yes	Yes	Yes	Yes
State Fixed Effects	Yes	Yes	Yes	Yes	Yes	Yes	Yes	Yes	Yes	Yes
State-specific Time Trend										
Linear	Yes	Yes	Yes	Yes	Yes	Yes	Yes	Yes	Yes	Yes
Quadratic		Yes	Yes	Yes	Yes		Yes	Yes	Yes	Yes
Cubic			Yes	Yes	Yes			Yes	Yes	Yes
Quartic				Yes	Yes				Yes	Yes
Quintic					Yes					Yes

Notes: Each column represents a separate regression using March CPS data. The dependent variable is the logarithm of hourly wage rate. In columns (1)-(5), we report OLS estimates of minimum wage on hourly wage (real in 2016 dollars) using the full sample. In columns (6)-(10), we report OLS estimates of minimum wage on hourly wage (real in 2016 dollars) using only low-skill sectors. We keep everyone with hourly wage less than 300% of federal (or state) minimum wage. The sample period is from 1990 through 2014. Control variables include age, sex, race (black and Hispanic indicators), education, married, and full time employed. Robust standard errors are clustered at the state level.

* significant at 5%

** significant at 1%.

The impact of the minimum wage for agriculture relative to other occupations is indicated by the coefficient on the interaction terms which are highly statistically significant and negative. This indicates that the experience of agriculture relative to other occupations is different and that minimum wage levels are economically irrelevant for agricultural workers whose coverage by the legislation may be limited. In other words, the impact of the minimum wage on wages received is greater within the nonagricultural population than in the agricultural worker population, and the magnitudes of the coefficients on minimum wage alone and when interacted with an agriculture dummy variable are roughly offsetting (indicated by α + γ). Since we are equating agriculture with its feature of limited minimum wage coverage, our findings are suggestive of the minimum wage environment having little impact on agricultural workers aggregately. Results are robust to the inclusion of state-specific time trends up to the fifth order to control for any time-varying state-specific heterogeneity. These results, however, may mask important differences within the agricultural worker population (for example, workers with different legal status). Furthermore, we cannot rule out impacts on other margins (such as intensive margins of work) without further examination. These features motivate our continued analysis using a specialized data set.

Since earnings are a function of both hourly wage and hours worked, we examine differences in hours worked per week in Table 3 to check for effects of the minimum wage along this intensive margin associated with work. Results are primarily null as statistically significant differences are not noted overall nor for agriculture relative to non-agricultural work.

**Table 3:** Minimum wage law and weekly hours.

	Full Sample					Low-Skill Sectors				
	(1)	(2)	(3)	(4)	(5)	(6)	(7)	(8)	(9)	(10)
Ln Minimum Wage	0.61	0.42	0.37	0.70	0.60	1.84*	1.35	1.05	0.89	0.43
	(0.48)	(0.51)	(0.56)	(0.65)	(0.63)	(0.90)	(0.99)	(1.26)	(1.53)	(1.49)
Ln Minimum Wage × Agriculture	3.32	3.26	3.28	3.30	3.29	2.30	2.21	2.32	2.49	2.52
	(2.18)	(2.18)	(2.17)	(2.18)	(2.18)	(2.14)	(2.14)	(2.11)	(2.11)	(2.14)
Agriculture	-6.48	-6.35	-6.38	-6.43	-6.42	-3.97	-3.80	-4.00	-4.35	-4.42
	(4.34)	(4.34)	(4.32)	(4.33)	(4.34)	(4.29)	(4.27)	(4.23)	(4.23)	(4.29)
α + γ	3.93	3.68	3.65	4.00	3.89	4.14	3.56	3.36	3.38	2.95
	(2.15)	(2.16)	(2.13)	(2.13)	(2.13)	(2.17)	(2.15)	(2.19)	(2.30)	(2.30)
Number of Observations	167,813	167,813	167,813	167,813	167,813	29,495	29,495	29,495	29,495	29,495
R-squared	0.67	0.67	0.67	0.67	0.67	0.68	0.68	0.68	0.68	0.68
Controls	Yes	Yes	Yes	Yes	Yes	Yes	Yes	Yes	Yes	Yes
Division-Year FE	Yes	Yes	Yes	Yes	Yes	Yes	Yes	Yes	Yes	Yes
State Fixed Effects	Yes	Yes	Yes	Yes	Yes	Yes	Yes	Yes	Yes	Yes
State-specific Time Trend										
Linear	Yes	Yes	Yes	Yes	Yes	Yes	Yes	Yes	Yes	Yes
Quadratic		Yes	Yes	Yes	Yes		Yes	Yes	Yes	Yes
Cubic			Yes	Yes	Yes			Yes	Yes	Yes
Quartic				Yes	Yes				Yes	Yes
Quintic					Yes					Yes

Notes: Each column represents a separate regression using March CPS data. The dependent variable is weekly hours. In columns (1)-(5), we report OLS estimates of minimum wage on weekly hours (real in 2016 dollars) using the full sample. In columns (6)-(10), we report OLS estimates of minimum wage on weekly hours (real in 2016 dollars) using only low-skill sectors. We keep everyone with hourly wage less than 300% of federal (or state) minimum wage. The sample period is from 1990 through 2014. Control variables include age, sex, race (black and Hispanic indicators), education, married, and full time employed. Robust standard errors are clustered at the state level.

* significant at 5%

** significant at 1%.

We conclude that primary differences accrue via changes to wages as opposed to the amount of work by this measure in terms of worker hours. Since our samples are comprised of workers alone and since the agricultural worker data particularly cannot be extended to include the unemployed due to its specific sample design, we do not address the extensive margin of work. Our results are not surprising given the nature of agricultural work and its feature of being very time sensitive (e.g., crops must be harvested on a fixed schedule). This also may be true of our comparison workers given the seasonality of construction and hospitality in many parts of the country.

### Robustness checks and extensions

We change the original assumption that the relevant sample is within only 300 percent of the minimum wage level in the supporting information (Panels A and B in S1 Table) where we consider the smaller samples of workers defined by 200 percent of the minimum wage level and 250 percent of this level respectively as a robustness exercise. By lowering the upper limit of the wage rate, we are able to focus most specifically on those workers for whom the minimum wage is potentially most binding because workers in the upper end of the distribution could plausibly be affected by the minimum wage solely through general equilibrium effects instead of direct effects of the wage itself. The findings are robust to these changes with statistically significant impacts of the minimum wage noted for wages but these impacts being offset for agricultural workers (as seen through the interaction term of interest).

We also compare the case of the United States to the subsample of the top 10 states in terms of total agricultural workers in Panel C of S1 Table. Due to sample size concerns, we do not examine the CPS data at the individual state level. Notably, more than 500 of the 1,153 total agricultural workers are sampled in 10 states. Sample sizes become too small to reliability examine individual states using these data. We find that our wage results are robust to this alternative sample division as well. This serves as additional motivation for the extension that we present for the case of the state of California drawing from specialized agricultural worker data.

We repeat our analysis for hours worked for each of the comparison samples in S2 Table. We again find insignificant differences in hours worked.

As a final extension, we consider fixed effects estimator triple differences which include state-time, time-occupation, and occupation-state differences (S3 Table). We aim to exploit the richness of the CPS data toward answering our primary policy questions regarding the effects of coverage of the minimum wages across several dimensions. Including state-by-time, time-by-coverage, and state-by-coverage fixed effects allows for the estimation of reliable estimates for ln(min_w_st_)×agriculture_ist_. Given these extra controls, we cannot estimate the coefficient on ln(min_w_st_) directly in this extension. We continue to find that agriculture has lesser effects of minimum wage changes than other occupational categories in terms of wages received and statistically insignificant differences in terms of hours worked.

Results using the CPS are both complementary to previous literature noted earlier in this paper. Papers from the 1970s on agricultural labor specifically found that that minimum wage coverage had a positive effect on wages but a negative impact on the size of the total agricultural labor force [10–11]. These papers, however, looked at the introduction of minimum wages over time as opposed to differences in minimum wages across time, space, and industry as we do here.

### National agricultural workers survey

Our data for the purpose of extending the analysis of farm workers for deeper examination come from the U.S. Department of Labor’s NAWS. The NAWS is a nationally representative dataset of employed U.S. farm workers which provides very specific information on hired field laborers. The data include detailed information on earnings and on socioeconomic characteristics broadly. Workers have been drawn from work sites three times per year since 1989. The public-use version of these data extends through 2014 and is what we use for this analysis in this paper.

Worksite-based sampling is notable because migrant workers may be undersampled in traditional residence-based surveys such as the CPS [36]. This could be a reason for the very small sample size for agricultural workers relative to other occupations in the CPS despite the large number of years from which we drew data. Another distinctive feature of NAWS is that H-2A workers (temporary nonimmigrant workers lawfully admitted performing temporary or seasonal agricultural work) are excluded from the respondent pool. While this may pose a disadvantage for some applications, this feature is attractive in this circumstance of this study since H-2A workers are protected by minimum wage legislation while other migrant workers are exempt. Since our focus is on the impact of lack of coverage, this exclusion allows us to better isolate the types of workers of most interest to our study question unlike what is possible with the CPS which presumably does include workers with H-2A visas. Finally, U.S. agriculture is characterized by a large percentage of unauthorized migrants, and unauthorized workers (who are identifiable in the NAWS but not in the CPS) may not receive wages above minimum levels. This feature offers another way to exploit these data to better understand relationships between minimum wage coverage and worker outcomes in agriculture.

In addition to being representative nationally, the NAWS is regionally representative for 12 geographic regions following areas determined by the U.S. Department of Agriculture to have similar agricultural characteristics. These are collapsed into six identifiable regions in the public-use version of the data. Since the regions are typically groupings of several states, the only state which is identifiable separately and able to be matched to minimum wage level data is California given its status as being a large producer in crop agriculture. We thus focus on the California sample as it can be cleanly matched to minimum wage data. Since California has implemented several state-level minimum wage increases over the sample period, this furthers the reasoning for focusing on this specific sub-sample.

Table 4 presents summary statistics of wages, hours, weeks, demographic characteristics, and work attributes for California NAWS workers in two main subcategories based on payment basis and one aggregate category. Hourly workers are those paid on a time-based schedule. Piece rate workers are paid according to their productivity. We present this in comparison to the full NAWS California sample.

**Table 4:** Summary statistics (national agricultural workers survey).

	Full Sample	Piece Rate Workers	Hourly Rate Workers
*Continuous Variables*			
Ln Hourly Earnings ($2016)	2.263	2.393	2.241
	(0.213)	(0.349)	(0.171)
Weekly Hours	43.89	37.77	44.89
	(11.38)	(10.54)	(11.20)
Age (Years)	35.15	33.44	35.43
	(11.61)	(11.27)	(11.64)
Education (Years)	6.231	5.783	6.304
	(3.355)	(3.271)	(3.363)
Farm Experience (Years)	12.24	10.99	12.45
	(9.531)	(8.756)	(9.637)
Job Tenure (Years)	5.318	4.080	5.521
	(5.471)	(3.999)	(5.651)
*Binary Variables (%)*			
Bonus Pay (= 1)	0.217	0.0845	0.239
Undocumented Workers	0.484	0.522	0.477
Female	0.192	0.172	0.195
Married	0.661	0.627	0.667
Speaks English	0.145	0.0945	0.153
Number of Observations	16,976	2,391	14,585

Notes: The sample period is from 1990 through 2014 to match the CPS sample period. Only workers in California are included in the sample. Workers from other states are not identifiable in the NAWS.

Observable differences across payment structures are notable on several dimensions. Hourly workers are on average almost two years older than are piece rate workers and are more likely to be married with spouses with them in the United States and to have higher rates of educational achievement (measured via years of schooling), U.S. farm work experience, years of tenure with their current employers, and self-reported English language abilities. Relatively low reported family sizes may be related to the age distribution of the migrant farm work population. Forty-eight percent of hourly workers report being unauthorized compared with 52 percent of piece rate workers.

Hourly equivalent wages are used for piece rate workers. This data construction uses information on average pay per unit and units per day (e.g., box, bin, etc.) along with crew size information when applicable). Piece rate workers are shown in Table 4 to realize higher wages per hour but work fewer hours per week on average consistent with past literature [35]. Piece rate workers are much less likely to report bonus payments, which may be an additional form of compensation for agricultural workers in the U.S. Much of the general literature on piece rate versus time rate payment has focused on how compensation structure affects both worker sorting across firms, and on incentives and productivity. Of particular interest is the common empirical finding that piece rate earnings distributions are characterized by a higher mean (consistent with theoretical predictions of increased effort), but larger variance than time rate earnings distributions [37–38]. Earnings premiums can be decomposed into compensating wage differential for increased risk (and thus increased income variation under piece rate contracts), and into incentive-effort effects since piece rate workers would exude more effort if they were compensated for it.

The piece rate sample is notably smaller with around 2,400 observations in comparison to more than 14,000 hourly rate workers. The vast differences between the characteristics of workers and their economic outcomes across the piece rate and hourly rate subsamples described in the table motivates the separate treatments of these workers in separate regressions in the estimation of Eq (2).

In addition to the theoretical stratification between piece rate and time rate workers in agriculture, workers are also divided in terms of legal coverage under minimum wage laws by their legal working status in the U.S. We use this feature to create an interaction term of interest to examine differential impacts of minimum wage within the farm worker population that we study.

Results from the estimation of Eq (2) utilizing the NAWS sample are presented in Table 5. We control for nonlinear time effects to control for common shocks that affects both undocumented and documented workers in California’s agricultural sector. Three outcome variables are considered: hourly wages, the presence of bonus payments (binary variable), and weekly hours worked. The regressions are presented for the full sample aggregating piece rate and hourly workers and then for these types of payment separately given their very different characteristics as indicated above in the discussion of the summary statistics.

**Table 5:** Minimum wage law and hourly wage, bonus, and weekly hours (NAWS-California).

	Ln Hourly Wage			Bonus			Weekly Hours		
	Full Sample	Piece Rate Workers	Hourly Rate Workers	Full Sample	Piece Rate Workers	Hourly Rate Workers	Full Sample	Piece Rate Workers	Hourly Rate Workers
	(1)	(2)	(3)	(4)	(5)	(6)	(7)	(8)	(9)
Ln Minimum Wage	0.31**	0.02	0.39**	-0.36**	-0.23	-0.43**	2.72	7.59	1.22
	(0.04)	(0.17)	(0.03)	(0.07)	(0.13)	(0.08)	(2.00)	(4.94)	(2.10)
Ln Minimum Wage × Unauthorized	0.16**	0.32	0.12**	0.17*	0.18	0.25**	-0.07	0.22	0.40
	(0.05)	(0.17)	(0.03)	(0.07)	(0.12)	(0.08)	(2.21)	(5.08)	(2.37)
Unauthorized	-0.37**	-0.73*	-0.28**	-0.44**	-0.41	-0.60**	-0.89	-1.97	-1.76
	(0.10)	(0.37)	(0.07)	(0.15)	(0.26)	(0.17)	(4.70)	(10.77)	(5.05)
α + γ	0.46**	0.34*	0.50**	-0.19**	-0.05	-0.18**	2.65	7.81	1.61
	(0.04)	(0.17)	(0.03)	(0.07)	(0.12)	(0.08)	(2.10)	(4.77)	(2.27)
Number of Observations	16,976	2,391	14,585	16,976	2,391	14,585	16,976	2,391	14,585
R-squared	0.12	0.12	0.21	0.16	0.10	0.17	0.07	0.04	0.08
Controls	Yes	Yes	Yes	Yes	Yes	Yes	Yes	Yes	Yes
Cubic Time Trend	Yes	Yes	Yes	Yes	Yes	Yes	Yes	Yes	Yes

Notes: Each column represents a separate regression using NAWS-California data. In columns (1)-(3), we report OLS estimates of minimum wage on hourly wage (real in 2016 dollars). In columns (4)-(6), we report OLS estimates of minimum wage on bonus (binary indicator of getting a bonus). In columns (7)-(9), we report OLS estimates of minimum wage on weekly hours. The sample period is from 1990 through 2014. Control variables include unauthorized status, age, sex, education, married, farm work experience, tenure years, and English. Robust standard errors are reported below the coefficients.

* significant at 5%

** significant at 1%.

The primary findings are that minimum wages have positive impacts on the wages of hourly workers who are covered directly by the legislation but do not have notable impacts statistically on the subsample of piece rate workers who are not all covered under minimum wage laws. Furthermore, workers who are unauthorized tend to realize greater gains from minimum wage legislation than do others. This correlation was initially unexpected but may relate to the growing scarcity of workers willing to work in crop agriculture when minimum wages are in place. As discussed in the conceptual framework earlier in this paper, the labor supply within the uncovered sector may decrease and the (uncovered) labor market may tighten as wages increase in the covered sector. This can create positive pressure on wage rates including those accruing to undocumented workers. If undocumented and documented workers are subject to separate labor markets (as in the dual labor market framework) and depending on differences in parameters across these markets, wages for undocumented workers may increase more than for documented workers. In addition, the patterns shown in Table 5 hypothetically may relate to the availability of migrant field labor overall in California if there are worker shortages inflating wages or may relate to institutional pressures to increase wages such as pressures stemming from California farmworker unions.

Since piece rate workers are much less likely to be formally covered by minimum wages, piece rate pay structure can be viewed as a proxy for the uncovered agricultural labor force and hourly pay for covered. The treatment of uncovered and covered parts of U.S. agriculture labor force is twofold in our results. First, piece rate and time rate payment structures are treated individually to allow separate estimations for the two coverage levels. Second, within these regressions, legal status is interacted with the treatment variable. If the assumption that piece rate status is a valid proxy for uncovered and hourly for covered employment, then the results in Table 5 suggest that the minimum wage has a positive impact on the wages of covered workers but little or no impact on the wages of uncovered workers in U.S. agriculture (where there are positive but statistically insignificant coefficients at the conventional level). This result was not evident from the analysis and adds to our understanding of the complexity of wages within the agricultural worker population (Table 5) as opposed to across the agricultural and non-agricultural sectors (Table 2). Our result is in contrast to Gindling and Terrell (2005) who find that minimum wages lead to substantial increases in wages received by both workers in formal large urban and rural enterprises and in informal small urban and rural enterprises where minimum wage laws are unenforced [39]. These authors, however, do not find a wage effect for the self-employed, suggesting that both context and type of employment matters.

Impacts on hours worked are statistically negligible for both types of workers, consistent with our findings in the analysis of CPS data earlier in this paper. Although drawn from different study populations, the results presented in Tables 2 and 5 are complementary and add to the breadth of our understanding of how minimum wages differentially affect subsets of the agricultural work community.

The NAWS data allows for the examination of bonus payments as an outcome variable to better understand payment dynamics. Similar data are not available in the CPS, and this represents another advantage on the extension analysis using NAWS. Since the specifications are implemented as linear probability models, we interpret the results as indicating that the probability of receiving a bonus payment decreases as the minimum wage becomes more generous. This negative impact, however, is muted for unauthorized workers who are paid on an hourly basis. This indicates bonuses and wages are more likely to be substitutes than complementary forms of compensation in agriculture. Depending on the (unobserved) size of payments relative to hourly wages, the net impact on worker income is ambiguous. We present results for bonus payments from an alternate specification using logit regression in S4 Table. Results are qualitatively similar.

## Discussion and conclusions

Farm workers have historically been among the poorest members of the U.S. working class, and limited work in agricultural labor economics to date has focused on specific policy to combat poverty in this population. Over the survey period, the federal minimum wage increased from $3.35 to $7.25 per hour, and some state minimum wages (including for the case of California which we exploit here) rose above the federally specified levels.

Results in this paper suggest that realized wages increase along with minimum wages for covered workers though this is not the case for workers with limited coverage, at least for the case study population of agriculture studied. Although there are negligible impacts on hours worked per week, negative total employment effects cannot be ruled out without further analysis. Orrenius and Zavodny (2008) and others find that minimum wages have not resulted in negative employment effects for adult immigrants with less than high school education [34]. Statistical evidence suggests that these workers may select to locate in areas with lower subnational minimum wages. The result also is consistent with Cadena’s (2014) model that predicts that immigrants following an objective to maximize earnings select locations with stagnant minimum wages (over states with increasing minimums) [40]. In agriculture, an immigrant-intensive industry where minimum wage coverage is not complete, welfare gains in terms of higher wages from extending minimum wages are plausibly limited to hourly paid workers which is another feature of our findings here.

Using multiple data sets for comparison and extension allows us to exploit stratification between agricultural and nonagricultural sectors, between piece rate and hourly workers, and between unauthorized and authorized workers in this study. We examine worker outcomes of hourly wages and hours worked per week in both the CPS and NAWS and can extend to bonus payments in the agricultural labor market analysis in order to derive further insights about substitutions of payment methods.

We draw our results from two sample surveys. There is evidence elsewhere in the literature that the CPS may have limits in terms of its representativeness of the U.S. farmworker population as it may undersample farm workers with some characteristics (notably those living in non-standard housing) [36]. The NAWS therefore has the advantage of allowance for migratory behavior in its original survey design. However, the NAWS itself has additional nuances relevant to the interpretation of the final results. The NAWS, for example, excludes H-2A workers whereas the CPS may include them. This is relevant due to strict regulations on the payment of H-2A workers which in some cases drive their wages higher than minimum levels. When this is the case, the minimum wage is less likely to be binding for these workers and thus the effects of minimum wages increases are less likely to be of practical significance. The inclusion of these workers in our CPS analysis may put a downward bias on the impact identified across agricultural and nonagricultural workers. On a practical level, this ultimately means that our two data sets are not representative of the same population and interpretations of our results in this paper need to be cognizant of these differences.

Overall, our paper contributes to understanding of the consequences of extending minimum wages to all U.S. farm workers. Results are relevant to discussions of the likely effects of new minimum wage increases, especially regarding industries where minimum wage coverage is incomplete. As noted, exemptions to the federal minimum wage extend beyond agriculture to, for example, workers with disabilities, full-time students, youth in their first days of consecutive employment, tipped employees, and student learners. Furthermore, workers in certain seasonal and recreational establishments and executive, administrative, professional, and outside sales employees who are paid on a salary basis are exempt as are some miscellaneous categories of other workers. Thus, farm workers are only one subgroup of U.S. workers for which minimum wages do not fully apply and results therefore may be relevant to policy discussions beyond agricultural markets. A caveat, as noted in the literature is that specific institutional and labor market characteristics (e.g., relative labor demand and supply elasticities across industries and sectors) may play important roles. Agriculture is distinctive due to its high proportion of unauthorized and seasonal immigrant workforce. The agricultural sector is often modeled in a dual labor market framework which complicates theoretical predictions of the impacts of changes to minimum wages and their coverage, though this provides justification and demand for the type of empirical modeling which we present.

Some authors have found positive distributional effects associated with minimum wages to be greater in rural as opposed to urban areas [41], and others find that employment effects of minimum wages may be spread over time and in the form of changes to employment growth rates [14]. While our study addresses hours worked per week and therefore employment indirectly on an intensive margin, we can say little about employment effects overall. Still, further examination of the effects of minimum wages on the extensive margin of work in agriculture versus other sectors is of policy importance especially given discussions of possible labor shortages in this sector. While our data structure had the characteristics of following workers and did not allow full examination of this margin, we encourage future study in this area to address this dimension as employment impacts associated with both covered and uncovered workers can affect optimal public policy response in terms of both labor markets, regulations and enforcement, and immigration and agricultural policy.

## Supporting information

S1 Table Minimum wage law and hourly wage (different cutoffs and top agricultural states). Each column represents a separate regression using March CPS data. We report OLS estimates of minimum wage on hourly wage (real in 2016 dollars) for the full sample and low-skill sectors. In Panel A, we keep everyone with hourly wage less than 200% of federal (or state) minimum wage. In Panel B, we keep everyone with hourly wage less than 250% of federal (or state) minimum wage. In Panel C, we restrict the sample to top 10 states with the most hired agricultural workers according to 2017 Census of Agriculture by USDA. The sample period is from 1990 through 2014. Control variables include age, sex, race (black and Hispanic indicators), education, married, and full time employed. Robust standard errors are clustered at the state level.
(DOCX)

S2 Table Minimum wage law and weekly Hhours (different cutoffs and top agricultural states). Each column represents a separate regression using March CPS data. We report OLS estimates of minimum wage on weekly hours for the full sample and low-skill sectors. In Panel A, we keep everyone with hourly wage less than 200% of federal (or state) minimum wage. In Panel B, we keep everyone with hourly wage less than 250% of federal (or state) minimum wage. In Panel C, we restrict the sample to top 10 states with the most hired agricultural workers according to 2017 Census of Agriculture by USDA. The sample period is from 1990 through 2014. Control variables include age, sex, race (black and Hispanic indicators), education, married, and full time employed. Robust standard errors are clustered at the state level.
(DOCX)

S3 Table Minimum wage law and hourly wage and weekly hours (FE estimator, triple differences). Each column represents a separate regression using March CPS data. In columns (1)-(3), we report OLS estimates of minimum wage on hourly wage (real in 2016 dollars). In columns (4)-(6), we report OLS estimates of minimum wage on weekly hours. We keep everyone with hourly wage less than 300% of federal (or state) minimum wage. The sample period is from 1990 through 2014. Control variables include age, sex, race (black and Hispanic indicators), education, married, and full time employed. Robust standard errors are clustered at the state level.
(DOCX)

S4 Table Minimum wage law and bonus (NAWS-California, logit). Each column represents a separate regression using NAWS-California data. We report Logit estimates of minimum wage on bonus (binary indicator of getting a bonus). The sample period is from 1990 through 2014. Control variables include unauthorized status, age, sex, education, married, farm work experience, tenure years, and English. Robust standard errors are reported below the coefficients.
(DOCX)

S1 File CPS search criteria. This file list all the variables we extracted from the Current Population Survey at the Minnesota Population Center. The access date is May 21^st^, 2017.
(PDF)
