# Supplementary material for: Do minimum wage laws affect those who are not covered? Evidence from agricultural and non-agricultural workers
Source: PLoS One. 2019 Oct 2;14(10):e0221935. doi: 10.1371/journal.pone.0221935 (PMC6774472; doi:10.1371/journal.pone.0221935)
Supplement: S1 Table — Each column represents a separate regression using March CPS data. We report OLS estimates of minimum wage on hourly wage (real in 2016 dollars) for the full sample and low-skill sectors. In Panel A, we keep everyone with hourly wage less than 200% of federal (or state) minimum wage. In Panel B, we keep everyone with hourly wage less than 250% of federal (or state) minimum wage. In Panel C, we restrict the sample to top 10 states with the most hired agricultural workers according to 2017 Census of Agriculture by USDA. The sample period is from 1990 through 2014. Control variables include age, sex, race (black and Hispanic indicators), education, married, and full time employed. Robust standard errors are clustered at the state level. (DOCX) [file pone.0221935.s001.docx]

S1 Table. Minimum Wage Law and Hourly Wage (Different Cutoffs and Top Agricultural States).

|  | Ln Hourly Wage | | | | | | |
| --- | --- | --- | --- | --- | --- | --- | --- |
|  | Full Sample | | |  | Low-Skill Sectors | | |
|  | (1) | (2) | (3) |  | (4) | (5) | (6) |
|  | Panel A: Below 200% Federal/State Minimum Wage | | | | | | |
| Ln Minimum Wage | 0.53** | 0.52** | 0.52** |  | 0.62** | 0.57** | 0.60** |
|  | (0.02) | (0.02) | (0.02) |  | (0.06) | (0.05) | (0.06) |
| Ln Minimum Wage × Agriculture | -0.42** | -0.42** | -0.42** |  | -0.62** | -0.62** | -0.62** |
|  | (0.07) | (0.07) | (0.07) |  | (0.12) | (0.12) | (0.12) |
| Agriculture | 0.78** | 0.78** | 0.77** |  | 1.23** | 1.23** | 1.22** |
|  | (0.15) | (0.15) | (0.15) |  | (0.25) | (0.24) | (0.25) |
| α + γ | 0.11 | 0.11 | 0.10 |  | 0.00 | -0.05 | -0.01 |
|  | (0.07) | (0.07) | (0.07) |  | (0.13) | (0.13) | (0.13) |
| Number of Observations | 109,865 | 109,865 | 109,865 |  | 22,842 | 22,842 | 22,842 |
| R-squared | 0.20 | 0.20 | 0.20 |  | 0.22 | 0.22 | 0.22 |
|  | Panel B: Below 250% Federal/State Minimum Wage | | | | | | |
| Ln Minimum Wage | 0.46** | 0.46** | 0.45** |  | 0.54** | 0.51** | 0.53** |
|  | (0.02) | (0.02) | (0.02) |  | (0.06) | (0.06) | (0.06) |
| Ln Minimum Wage × Agriculture | -0.41** | -0.41** | -0.41** |  | -0.58** | -0.58** | -0.58** |
|  | (0.08) | (0.08) | (0.08) |  | (0.13) | (0.13) | (0.13) |
| Agriculture | 0.70** | 0.70** | 0.70** |  | 1.09** | 1.10** | 1.09** |
|  | (0.16) | (0.16) | (0.16) |  | (0.25) | (0.25) | (0.26) |
| α + γ | 0.05 | 0.05 | 0.04 |  | -0.04 | -0.07 | -0.05 |
|  | (0.08) | (0.08) | (0.08) |  | (0.14) | (0.15) | (0.15) |
| Number of Observations | 140,611 | 140,611 | 140,611 |  | 26,180 | 26,180 | 26,180 |
| R-squared | 0.23 | 0.23 | 0.23 |  | 0.26 | 0.26 | 0.27 |
|  | Panel C: Top 10 States Hiring the Most Agricultural Workers | | | | | | |
| Ln Minimum Wage | 0.51** | 0.51** | 0.49** |  | 0.82** | 0.80** | 0.80** |
|  | (0.06) | (0.06) | (0.06) |  | (0.12) | (0.12) | (0.13) |
| Ln Minimum Wage × Agriculture | -0.33* | -0.34* | -0.34* |  | -0.62** | -0.61** | -0.61** |
|  | (0.11) | (0.11) | (0.11) |  | (0.13) | (0.13) | (0.13) |
| Agriculture | 0.49 | 0.49 | 0.50 |  | 1.10** | 1.09** | 1.09** |
|  | (0.23) | (0.23) | (0.22) |  | (0.27) | (0.27) | (0.27) |
|  | 1.01** | 1.00** | 1.05** |  | 0.32 | 0.35 | 0.36 |
|  | (0.13) | (0.13) | (0.13) |  | (0.22) | (0.23) | (0.25) |
| α + γ | 0.17 | 0.18 | 0.15 |  | 0.21 | 0.19 | 0.19 |
|  | (0.13) | (0.13) | (0.12) |  | (0.18) | (0.19) | (0.19) |
| Number of Observations | 51,813 | 51,813 | 51,813 |  | 9,277 | 9,277 | 9,277 |
| R-squared | 0.26 | 0.26 | 0.26 |  | 0.29 | 0.29 | 0.29 |
| Controls | Yes | Yes | Yes |  | Yes | Yes | Yes |
| Division-Year FE | Yes | Yes | Yes |  | Yes | Yes | Yes |
| State Fixed Effects | Yes | Yes | Yes |  | Yes | Yes | Yes |
| State-specific Time Trend |  |  |  |  |  |  |  |
| Linear | Yes | Yes | Yes |  | Yes | Yes | Yes |
| Quadratic |  | Yes | Yes |  |  | Yes | Yes |
| Cubic |  |  | Yes |  |  |  | Yes |

Notes: Each column represents a separate regression using March CPS data. We report OLS estimates of minimum wage on hourly wage (real in 2016 dollars) for the full sample and low-skill sectors. In Panel A, we keep everyone with hourly wage less than 200% of federal (or state) minimum wage. In Panel B, we keep everyone with hourly wage less than 250% of federal (or state) minimum wage. In Panel C, we restrict the sample to top 10 states with the most hired agricultural workers according to 2017 Census of Agriculture by USDA. The sample period is from 1990 through 2014. Control variables include age, sex, race (black and Hispanic indicators), education, married, and full time employed. Robust standard errors are clustered at the state level. * significant at 5% ** significant at 1%.
