# Supplementary material for: Do minimum wage laws affect those who are not covered? Evidence from agricultural and non-agricultural workers
Source: PLoS One. 2019 Oct 2;14(10):e0221935. doi: 10.1371/journal.pone.0221935 (PMC6774472; doi:10.1371/journal.pone.0221935)
Supplement: S3 Table — Each column represents a separate regression using March CPS data. In columns (1)-(3), we report OLS estimates of minimum wage on hourly wage (real in 2016 dollars). In columns (4)-(6), we report OLS estimates of minimum wage on weekly hours. We keep everyone with hourly wage less than 300% of federal (or state) minimum wage. The sample period is from 1990 through 2014. Control variables include age, sex, race (black and Hispanic indicators), education, married, and full time employed. Robust standard errors are clustered at the state level. (DOCX) [file pone.0221935.s003.docx]

S3 Table. Minimum Wage Law and Hourly Wage and Weekly Hours (FE Estimator, Triple Differences).

|  | Ln Hourly Wage | | | |  | Weekly Hours | | | |
| --- | --- | --- | --- | --- | --- | --- | --- | --- | --- |
|  | Full Sample | |  | Low-Skill Sectors |  | Full Sample | |  | Low-Skill Sectors |
|  | (1) | (2) |  | (3) |  | (4) | (5) |  | (6) |
| Ln Minimum Wage × Agriculture | -0.55** | -0.51** |  | -0.45* |  | 3.25 | -1.56 |  | 0.78 |
|  | (0.13) | (0.11) |  | (0.18) |  | (6.22) | (3.70) |  | (4.34) |
| Agriculture | 1.00** | 1.01** |  | 1.06** |  | -12.74 | 1.23 |  | -2.33 |
|  | (0.26) | (0.22) |  | (0.37) |  | (13.73) | (7.73) |  | (8.88) |
| Number of Observations | 161,729 | 161,729 |  | 28,406 |  | 167,813 | 167,813 |  | 29,495 |
| R-squared | 0.05 | 0.26 |  | 0.33 |  | 0.02 | 0.68 |  | 0.69 |
| Controls | No | Yes |  | Yes |  | No | Yes |  | Yes |
| Year FE | Yes | Yes |  | Yes |  | Yes | Yes |  | Yes |
| State FE | Yes | Yes |  | Yes |  | Yes | Yes |  | Yes |
| State-Year FE | Yes | Yes |  | Yes |  | Yes | Yes |  | Yes |
| Agriculture-State FE | Yes | Yes |  | Yes |  | Yes | Yes |  | Yes |
| Agriculture-Year FE | Yes | Yes |  | Yes |  | Yes | Yes |  | Yes |

Notes: Each column represents a separate regression using March CPS data. In columns (1)-(3), we report OLS estimates of minimum wage on hourly wage (real in 2016 dollars). In columns (4)-(6), we report OLS estimates of minimum wage on weekly hours. We keep everyone with hourly wage less than 300% of federal (or state) minimum wage. The sample period is from 1990 through 2014. Control variables include age, sex, race (black and Hispanic indicators), education, married, and full time employed. Robust standard errors are clustered at the state level. * significant at 5% ** significant at 1%.
