# Supplementary material for: Do minimum wage laws affect those who are not covered? Evidence from agricultural and non-agricultural workers
Source: PLoS One. 2019 Oct 2;14(10):e0221935. doi: 10.1371/journal.pone.0221935 (PMC6774472; doi:10.1371/journal.pone.0221935)
Supplement: S4 Table — Each column represents a separate regression using NAWS-California data. We report Logit estimates of minimum wage on bonus (binary indicator of getting a bonus). The sample period is from 1990 through 2014. Control variables include unauthorized status, age, sex, education, married, farm work experience, tenure years, and English. Robust standard errors are reported below the coefficients. (DOCX) [file pone.0221935.s004.docx]

S4 Table. Minimum Wage Law and Bonus (NAWS-California, Logit).

|  | Full Sample | Piece Rate Workers | Hourly Rate Workers |
| --- | --- | --- | --- |
|  | (1) | (2) | (3) |
|  | Coefficients | | |
| Ln Minimum Wage | -2.97** | -5.70** | -3.19** |
|  | (0.44) | (1.76) | (0.47) |
| Ln Minimum Wage × Unauthorized | 2.96** | 2.06 | 3.40** |
|  | (0.64) | (1.95) | (0.70) |
| Unauthorized | -6.89** | -4.79 | -7.83** |
|  | (1.37) | (4.11) | (1.49) |
|  | Marginal Effects | | |
| Ln Minimum Wage (at Mean) | 0.18** | 0.04** | 0.20** |
|  | (0.01) | (0.01) | (0.01) |
| Unauthorized (=1) | 0.17** | 0.06** | 0.19** |
|  | (0.00) | (0.01) | (0.01) |
| Unauthorized (=0) | 0.25** | 0.10** | 0.27** |
|  | (0.00) | (0.01) | (0.01) |
| Number of Observations | 16,976 | 2,391 | 14,585 |
| Controls | Yes | Yes | Yes |
| Cubic Time Trend | Yes | Yes | Yes |

Notes: Each column represents a separate regression using NAWS-California data. We report Logit estimates of minimum wage on bonus (binary indicator of getting a bonus). The sample period is from 1990 through 2014. Control variables include unauthorized status, age, sex, education, married, farm work experience, tenure years, and English. Robust standard errors are reported below the coefficients. * significant at 5% ** significant at 1%.
