# Supplementary material for: Do minimum wage laws affect those who are not covered? Evidence from agricultural and non-agricultural workers
Source: PLoS One. 2019 Oct 2;14(10):e0221935. doi: 10.1371/journal.pone.0221935 (PMC6774472; doi:10.1371/journal.pone.0221935)
Supplement: S1 File — This file list all the variables we extracted from the Current Population Survey at the Minnesota Population Center. The access date is May 21st, 2017. (PDF) [file pone.0221935.s005.pdf]

MPC Minnesota Population Center

User Extract cps_00016.dat

Jump to Section

1. Document Description
2. Study Description
3. File Description
4. Variable Description

§ 1. Document Description

Citation

**Title Statement**	
Title:	Codebook for an IPUMS-CPS Data Extract
Subtitle:	DDI 2.1 metadata describing the extract file 'cps_00016.dat'
Identification Number:	ddi2-69109_cps_00016.dat-cps.ipums.org
**Responsibility Statement**	
Authoring Entity:	Minnesota Population Center
Affiliation:	University of Minnesota
**Production Statement**	
Producer:	Minnesota Population Center
Affiliation:	University of Minnesota
Role:	Documentation
Date of Production:	May 21, 2017
Place of Production:	Minnesota Population Center, 50 Willey Hall, 225 - 19th Avenue South, Minneapolis, MN 55455

Distribution Statement	
Contact Persons:	Minnesota Population Center
Affiliation:	University of Minnesota
URI:	http://pop.umn.edu

§ 2. Study Description

Citation

Title Statement	
Title:	User Extract cps_00016.dat
**Responsibility Statement**	
Authoring Entity:	Minnesota Population Center
Affiliation:	University of Minnesota
**Production Statement**	
Producer:	Minnesota Population Center
Affiliation:	University of Minnesota
Role:	Documentation
Date of Production:	May 21, 2017
Place of Production:	Minnesota Population Center, 50 Willey Hall, 225 - 19th Avenue South, Minneapolis, MN 55455
**Distribution Statement**	
Contact Persons:	Minnesota Population Center
Affiliation:	University of Minnesota
URI:	http://pop.umn.edu
**Version Statement**	

Date:	2017-05-21

Study Scope

Subject Information	
Topic Classification:	Technical Variables -- HOUSEHOLD
	Linking Variables -- HOUSEHOLD
	Geographic Variables -- HOUSEHOLD
	Technical Variables -- PERSON
	Linking Variables -- PERSON
	Family Interrelationship Variables -- PERSON
	Core Demographic Variables -- PERSON
	Ethnicity/Nativity Variables -- PERSON
	Education Variables -- PERSON
	Work Variables -- PERSON
	Outgoing Rotation Groups (Earner Study) Variables -- PERSON
	Income Variables -- PERSON
**Summary Data Description**	
Time Period:	1980-03
Country:	United States
**Summary Data Description**	
Time Period:	1981-03
Country:	United States
**Summary Data Description**	
Time Period:	1982-03

Country:	United States
**Summary Data Description**	
Time Period:	1983-03
Country:	United States
**Summary Data Description**	
Time Period:	1984-03
Country:	United States
**Summary Data Description**	
Time Period:	1985-03
Country:	United States
**Summary Data Description**	
Time Period:	1986-03
Country:	United States
**Summary Data Description**	
Time Period:	1987-03
Country:	United States
**Summary Data Description**	
Time Period:	1988-03
Country:	United States
**Summary Data Description**	
Time Period:	1989-03
Country:	United States
**Summary Data Description**	

Time Period:	1990-03
Country:	United States
**Summary Data Description**	
Time Period:	1991-03
Country:	United States
**Summary Data Description**	
Time Period:	1992-03
Country:	United States
**Summary Data Description**	
Time Period:	1993-03
Country:	United States
**Summary Data Description**	
Time Period:	1994-03
Country:	United States
**Summary Data Description**	
Time Period:	1995-03
Country:	United States
**Summary Data Description**	
Time Period:	1996-03
Country:	United States
**Summary Data Description**	
Time Period:	1997-03
Country:	United States

**Summary Data Description**	
Time Period:	1998-03
Country:	United States
**Summary Data Description**	
Time Period:	1999-03
Country:	United States
**Summary Data Description**	
Time Period:	2000-03
Country:	United States
**Summary Data Description**	
Time Period:	2001-03
Country:	United States
**Summary Data Description**	
Time Period:	2002-03
Country:	United States
**Summary Data Description**	
Time Period:	2003-03
Country:	United States
**Summary Data Description**	
Time Period:	2004-03
Country:	United States
**Summary Data Description**	
Time Period:	2005-03

Country:	United States
**Summary Data Description**	
Time Period:	2006-03
Country:	United States
**Summary Data Description**	
Time Period:	2007-03
Country:	United States
**Summary Data Description**	
Time Period:	2008-03
Country:	United States
**Summary Data Description**	
Time Period:	2009-03
Country:	United States
**Summary Data Description**	
Time Period:	2010-03
Country:	United States
**Summary Data Description**	
Time Period:	2011-03
Country:	United States
**Summary Data Description**	
Time Period:	2012-03
Country:	United States
**Summary Data Description**	

Time Period:	2013-03
Country:	United States
**Summary Data Description**	
Time Period:	2014-03
Country:	United States
**Summary Data Description**	
Time Period:	2015-03
Country:	United States
**Summary Data Description**	
Time Period:	2016-03
Country:	United States
**Notes**	
Note:	Additional notes on a sample that is part of this study: IPUMS-CPS, ASEC 1980; regular size
	Additional notes on a sample that is part of this study: IPUMS-CPS, ASEC 1981; regular size
	Additional notes on a sample that is part of this study: IPUMS-CPS, ASEC 1982; regular size
	Additional notes on a sample that is part of this study: IPUMS-CPS, ASEC 1983; regular size
	Additional notes on a sample that is part of this study: IPUMS-CPS, ASEC 1984; regular size
	Additional notes on a sample that is part of this study: IPUMS-CPS, ASEC 1985; regular size
	Additional notes on a sample that is part of this study: IPUMS-CPS, ASEC 1986; regular size
	Additional notes on a sample that is part of this study: IPUMS-CPS, ASEC 1987; regular size

	Additional notes on a sample that is part of this study: IPUMS-CPS, ASEC 1988; regular size
	Additional notes on a sample that is part of this study: IPUMS-CPS, ASEC 1989; regular size
	Additional notes on a sample that is part of this study: IPUMS-CPS, ASEC 1990; regular size
	Additional notes on a sample that is part of this study: IPUMS-CPS, ASEC 1991; regular size
	Additional notes on a sample that is part of this study: IPUMS-CPS, ASEC 1992; regular size
	Additional notes on a sample that is part of this study: IPUMS-CPS, ASEC 1993; regular size
	Additional notes on a sample that is part of this study: IPUMS-CPS, ASEC 1994; regular size
	Additional notes on a sample that is part of this study: IPUMS-CPS, ASEC 1995; regular size
	Additional notes on a sample that is part of this study: IPUMS-CPS, ASEC 1996; regular size
	Additional notes on a sample that is part of this study: IPUMS-CPS, ASEC 1997; regular size
	Additional notes on a sample that is part of this study: IPUMS-CPS, ASEC 1998; regular size
	Additional notes on a sample that is part of this study: IPUMS-CPS, ASEC 1999; regular size
	Additional notes on a sample that is part of this study: IPUMS-CPS, ASEC 2000; regular size
	Additional notes on a sample that is part of this study: IPUMS-CPS, ASEC 2001; regular size
	Additional notes on a sample that is part of this study: IPUMS-CPS, ASEC 2002; regular size
	Additional notes on a sample that is part of this study: IPUMS-CPS, ASEC 2003; regular size
	Additional notes on a sample that is part of this study: IPUMS-CPS, ASEC 2004; regular size

	Additional notes on a sample that is part of this study: IPUMS-CPS, ASEC 2005; regular size
	Additional notes on a sample that is part of this study: IPUMS-CPS, ASEC 2006; regular size
	Additional notes on a sample that is part of this study: IPUMS-CPS, ASEC 2007; regular size
	Additional notes on a sample that is part of this study: IPUMS-CPS, ASEC 2008; regular size
	Additional notes on a sample that is part of this study: IPUMS-CPS, ASEC 2009; regular size
	Additional notes on a sample that is part of this study: IPUMS-CPS, ASEC 2010; regular size
	Additional notes on a sample that is part of this study: IPUMS-CPS, ASEC 2011; regular size
	Additional notes on a sample that is part of this study: IPUMS-CPS, ASEC 2012; regular size
	Additional notes on a sample that is part of this study: IPUMS-CPS, ASEC 2013; regular size
	Additional notes on a sample that is part of this study: IPUMS-CPS, ASEC 2014; regular size
	Additional notes on a sample that is part of this study: IPUMS-CPS, ASEC 2015; regular size
	Additional notes on a sample that is part of this study: IPUMS-CPS, ASEC 2016; regular size

Data Access - Use Statement

**Confidentiality Declaration**	
None	
Contact Persons:	IPUMS-CPS
Affiliation:	Minnesota Population Center
URI:	http://cps.ipums.org/
**Citation Requirement**	

Publications and research reports based on the IPUMS-CPS database must cite it appropriately. The citation should include the following:

Sarah Flood, Miriam King, Steven Ruggles, and J. Robert Warren. <i>Integrated Public Use Microdata Series, Current Population Survey: Version 4.0</i> [dataset]. Minneapolis, MN: University of Minnesota, 2015.
http://doi.org/10.18128/D030.V4.0.

The licensing agreement for use of IPUMS-CPS data requires that users supply us with the title and full citation for any publications, research reports, or educational materials making use of the data or documentation. Please add your citation to the IPUMS bibliography: http://bibliography.ipums.org/

Conditions

Users of IPUMS-CPS data must agree to abide by the conditions of use. A user's license is valid for one year and may be renewed. Users must agree to the following conditions:

(1) No fees may be charged for use or distribution of the data. All persons are granted a limited license to use these data, but you may not charge a fee for the data if you distribute it to others.

(2) Cite IPUMS appropriately. For information on proper citation, refer to the citation requirement section of this DDI document.

(3) Tell us about any work you do using the IPUMS. Publications, research reports, or presentations making use of IPUMS-CPS should be added to our Bibliography. Continued funding for the IPUMS depends on our ability to show our sponsor agencies that researchers are using the data for productive purposes.

(4) Use it for GOOD -- never for EVIL.

Disclaimer

The user of the data acknowledges that the original collector of the data, the authorized distributor of the data, and the relevant funding agency bear no responsibility for use of the data or for interpretations or inferences based upon such uses.

Study Notes

**Notes**	
Note:	User-provided description: Revision of (Revision of (Revision of (Revision of (CPS_minimum_Wage1))))
	This extract is a revision of the user's previous extract, number 15.

§ 3. File Description

File

File Name:	cps_00016.dat
Contents of Files:	Microdata records

Type:	rectangular
File Type:	ISO-8859-1 data file
Data Format:	fixed length fields
Place of File Production:	Minnesota Population Center, 50 Willey Hall, 225 - 19th Avenue South, Minneapolis, MN 55455

§ 4. Variable Description

Jump to Variable

1. YEAR (Survey year)
2. SERIAL (Household serial number)
3. HWTSUPP (Household weight, Supplement)
4. CPSID (CPSID, household record)
5. STATEFIP (State (FIPS code))
6. STATECENSUS (State (Census code))
7. ASECFLAG (Flag for ASEC)
8. HFLAG (Flag for the 3/8 file 2014)
9. METRO (Metropolitan central city status)
10. COUNTY (FIPS county code)
11. METFIPS (Metropolitan area FIPS code)
12. CPI99 (CPI-U adjustment factor to 1999 dollars)
13. MONTH (Month)
14. PERNUM (Person number in sample unit)
15. CPSIDP (CPSID, person record)
16. WTSUPP (Supplement Weight)
17. EARNWT (Earnings weight)
18. NCHILD (Number of own children in household)
19. AGE (Age)
20. SEX (Sex)
21. RACE (Race)
22. MARST (Marital status)
23. POPSTAT (Adult civilian, armed forces, or child)
24. BPL (Birthplace)
25. YRIMMIG (Year of immigration)
26. CITIZEN (Citizenship status)
27. NATIVITY (Foreign birthplace or parentage)
28. HISPAN (Hispanic origin)
29. EDUC (Educational attainment recode)
30. EDUC99 (Educational attainment, 1990)
31. EMPSTAT (Employment status)
32. OCC (Occupation)

33. OCC2010 (Occupation, 2010 basis)
34. OCC1990 (Occupation, 1990 basis)
35. IND1990 (Industry, 1990 basis)
36. OCC1950 (Occupation, 1950 basis)
37. IND (Industry)
38. IND1950 (Industry, 1950 basis)
39. CLASSWKR (Class of worker)
40. WKSWORK1 (Weeks worked last year)
41. UHRSWORKLY (Usual hours worked per week (last yr))
42. UHRSWORKT (Hours usually worked per week at all jobs)
43. UHRSWORK1 (Hours usually worked per week at main job)
44. AHRSWORKT (Hours worked last week)
45. HOURWAGE (Hourly wage)
46. PAIDHOUR (Paid by the hour)
47. UNION (Union membership)
48. FIRMSIZE (Number of employees)
49. INCWAGE (Wage and salary income)
50. INCFARM (Farm income)
51. EARNWEEK (Weekly earnings)
52. OINCWAGE (Earnings from other work included wage and salary earnings)
53. WKSTAT (Full or part time status)

Variable: "YEAR"

Name:	YEAR
Label:	Survey year
Variable Text:	YEAR reports the year in which the survey was conducted. YEARP is repeated on person records.
Concept:	Technical Variables -- HOUSEHOLD
Start Position:	1
End Position:	4
Width:	4
Variable Format:	numeric
Implied Decimal Places:	0
Coder Instructions:	YEAR is a 4-digit numeric value.

Variable: "SERIAL"

Name:	SERIAL
Label:	Household serial number
Variable Text:	SERIAL is an identifying number unique to each household in a given survey month and year. All person records are assigned the same serial number as the household record they follow. A combination of YEAR, MONTH, and SERIAL provides a within-sample unique identifier for every household in IPUMS-CPS; YEAR, SERIAL, and PERNUM uniquely identify every person in the database within sample.<br><br>SERIAL is a new value generated for IPUMS-CPS and should not be confused with the household serial number created by the Census Bureau and included in the original CPS data.
Concept:	Technical Variables -- HOUSEHOLD
Start Position:	5
End Position:	9
Width:	5
Variable Format:	numeric
Implied Decimal Places:	0
Coder Instructions:	SERIAL is a 5-digit numeric variable.

Variable: "HWTSUPP"

Name:	HWTSUPP
Label:	Household weight, Supplement
Variable Text:	HWTSUPP is a household-level weight that should be used to generate statistics about households in March Annual Social and Economic (ASEC) Supplement data. The CPS uses a complex stratified sampling scheme, and HWTSUPP must be used to produce unbiased household-level statistics from the IPUMS-CPS ASEC data. For analyses of non-ASEC data, researchers should use HWTFINL. For individual-level analyses, researchers should use WTFINL, WTSUPP, or EARNWT.<br><br>HWTSUPP generally has the same value as WTSUPP for the household head or reference person. Vacant housing units and households that could not be interviewed due to residents' absence or refusal to participate have a value of zero in HWTSUPP; such sampled units were included in the public use CPS data beginning in 1988.

	Estimates on the entire population are prepared by projecting forward the resident population from the last available census. These projections are derived by updating the demographic census data from a number of other data sources that account for death, births and net migration. About 3 years after every census (i.e. 2003 for the 2000 Census and 2013 for the 2010 Census), the Census Bureau updates its independent population control and provides a new weight for the relevant years.<br><br>Two important points should be noted here. First, the lag between when the Census is conducted and when the CPS weights are updated is about 3 years. While the Census data are being processed, the CPS files are made available using the weighting scheme from the US Census prior to the latest Census. Second, once the files are updated, the old weights become obsolete and are replaced in the IPUMS data extract system. Published estimates from the lag years that use the old weights are not always updated. For example, 2010 poverty estimates were released in ASEC using the 2000 population controls. Once the 2010 population controls were made available, IPUMS-CPS replaced the ASEC 2010, 2011, and 2012 weights that are based on the 2000 population control with weights that are based on the 2010 population controls.<br><br>IPUMS-CPS makes available only the most up-to-date weights. The old values are available here: Old SPM and Weights Values [URL omitted from DDI.].
Concept:	Technical Variables -- HOUSEHOLD
Start Position:	10
End Position:	19
Width:	10
Variable Format:	numeric
Implied Decimal Places:	4
Coder Instructions:	HWTSUPP is a 10-digit numeric variable with four implied decimals. That is, 1234567890 should be interpreted as 123456.7890. The IPUMS command files automatically divide HWTSUPP by 10,000, so no further adjustment is needed.

Variable: "CPSID"

Name:	CPSID
Label:	CPSID, household record
Variable Text:	CPSID is an IPUMS-CPS defined variable that uniquely identifies households across CPS samples. The first six digits of CPSID index the four-digit year and two-digit month that the household was first in the CPS. CPSID allows users to link a household record across samples, based on the 4-8-4 rotation pattern, by assigning a unique CPSID value based on a combination of household identifiers. CPSID will only ever appear for a maximum of 8 times, which is the number of times a household may be

	observed in the CPS survey (as indexed by MIS). In some cases, a household will appear fewer than 8 times due to migration, mortality, non-response, and recording errors. CPSID Extensive documentation about the creation of CPSID is available elsewhere [URL omitted from DDI.].<br><br>CPSID may also be used to link ASEC respondents who are in the March Basic Monthly file to other months of CPS data. This linking is made possible by IPUMS through the creation of MARBASECIDP. Users should note that ASEC oversample households (as indicated by ASECOVERH) will always have a CPSID value of 0.<br><br>Users may also want to see CPSIDP for more information about linking individuals across time using a person-specific version of CPSID.
Concept:	Linking Variables -- HOUSEHOLD
Start Position:	20
End Position:	33
Width:	14
Variable Format:	numeric
Implied Decimal Places:	0
Coder Instructions:	CPSID is a 14-digit numeric variable.

Variable: "STATEFIP"

Name:	STATEFIP
Label:	State (FIPS code)
Variable Text:	STATEFIP identifies the household's state of residence, using the Federal Information Processing Standards (FIPS) coding scheme, which orders the states alphabetically.
Concept:	Geographic Variables -- HOUSEHOLD
Start Position:	34
End Position:	35
Width:	2
Variable Format:	numeric

Implied Decimal Places:	0

Categories

Value	Label
01	Alabama
02	Alaska
04	Arizona
05	Arkansas
06	California
08	Colorado
09	Connecticut
10	Delaware
11	District of Columbia
12	Florida
13	Georgia
15	Hawaii
16	Idaho
17	Illinois
18	Indiana
19	Iowa
20	Kansas
21	Kentucky
22	Louisiana
23	Maine

24	Maryland
25	Massachusetts
26	Michigan
27	Minnesota
28	Mississippi
29	Missouri
30	Montana
31	Nebraska
32	Nevada
33	New Hampshire
34	New Jersey
35	New Mexico
36	New York
37	North Carolina
38	North Dakota
39	Ohio
40	Oklahoma
41	Oregon
42	Pennsylvania
44	Rhode Island
45	South Carolina
46	South Dakota
47	Tennessee
48	Texas

49	Utah
50	Vermont
51	Virginia
53	Washington
54	West Virginia
55	Wisconsin
56	Wyoming
61	Maine-New Hampshire-Vermont
65	Montana-Idaho-Wyoming
68	Alaska-Hawaii
69	Nebraska-North Dakota-South Dakota
70	Maine-Massachusetts-New Hampshire-Rhode Island-Vermont
71	Michigan-Wisconsin
72	Minnesota-Iowa
73	Nebraska-North Dakota-South Dakota-Kansas
74	Delaware-Virginia
75	North Carolina-South Carolina
76	Alabama-Mississippi
77	Arkansas-Oklahoma
78	Arizona-New Mexico-Colorado
79	Idaho-Wyoming-Utah-Montana-Nevada
80	Alaska-Washington-Hawaii
81	New Hampshire-Maine-Vermont-Rhode Island
83	South Carolina-Georgia

84	Kentucky-Tennessee
85	Arkansas-Louisiana-Oklahoma
87	Iowa-N Dakota-S Dakota-Nebraska-Kansas-Minnesota-Missouri
88	Washington-Oregon-Alaska-Hawaii
89	Montana-Wyoming-Colorado-New Mexico-Utah-Nevada-Arizona
90	Delaware-Maryland-Virginia-West Virginia
99	State not identified

Variable: "STATECENSUS"

Name:	STATECENSUS
Label:	State (Census code)
Variable Text:	STATECENSUS identifies the household's state of residence using Census state codes.
Concept:	Geographic Variables -- HOUSEHOLD
Start Position:	36
End Position:	37
Width:	2
Variable Format:	numeric
Implied Decimal Places:	0

Categories

Value	Label
00	Unknown
11	Maine

12	New Hampshire
13	Vermont
14	Massachusetts
15	Rhode Island
16	Connecticut
19	Maine, New Hampshire, Vermont, Rhode Island
21	New York
22	New Jersey
23	Pennsylvania
31	Ohio
32	Indiana
33	Illinois
34	Michigan
35	Wisconsin
39	Michigan, Wisconsin
41	Minnesota
42	Iowa
43	Missouri
44	North Dakota
45	South Dakota
46	Nebraska
47	Kansas
49	Minnesota, Iowa, Missouri, North Dakota, South Dakota, Nebraska, Kansas
50	Delaware, Maryland, Virginia, West Virginia

51	Delaware
52	Maryland
53	District of Columbia
54	Virginia
55	West Virginia
56	North Carolina
57	South Carolina
58	Georgia
59	Florida
60	South Carolina, Georgia
61	Kentucky
62	Tennessee
63	Alabama
64	Mississippi
67	Kentucky, Tennessee
69	Alabama, Mississippi
71	Arkansas
72	Louisiana
73	Oklahoma
74	Texas
79	Arkansas, Louisiana, Oklahoma
81	Montana
82	Idaho
83	Wyoming

84	Colorado
85	New Mexico
86	Arizona
87	Utah
88	Nevada
89	Montana, Idaho, Wyoming, Colorado, New Mexico, Arizona, Utah, Nevada
91	Washington
92	Oregon
93	California
94	Alaska
95	Hawaii
99	Washington, Oregon, Alaska, Hawaii

Variable: "ASECFLAG"

Name:	ASECFLAG
Label:	Flag for ASEC
Variable Text:	ASECFLAG indicates whether the respondent is part of the ASEC or the March Basic. This variable is useful for users who wish to distinguish ASEC and March Basic files in their extracts. See further information [URL omitted from DDI.] about the ASEC versus the March Basic Monthly Files.
Concept:	Technical Variables -- HOUSEHOLD
Start Position:	38
End Position:	38
Width:	1
Variable Format:	numeric

Implied Decimal Places:	0

Categories

Value	Label
1	ASEC
2	March Basic

Variable: "HFLAG"

Name:	HFLAG
Label:	Flag for the 3/8 file 2014
Variable Text:	HFLAG indicates whether the respondent is part of the 3/8 redesign in the 2014 ASEC sample. This variable is useful for users who wish to analyze income and SPM variables in the 2014 ASEC sample. See further information [URL omitted from DDI.] about the 2014 survey redesign.
Concept:	Technical Variables -- HOUSEHOLD
Start Position:	39
End Position:	39
Width:	1
Variable Format:	numeric
Implied Decimal Places:	0

Categories

Value	Label
0	5/8 file

1	3/8 file

Variable: "METRO"

Name:	METRO
Label:	Metropolitan central city status
Variable Text:	METRO indicates whether a household was located in a metropolitan area. For households within metropolitan areas, METRO specifies whether the housing unit was inside or outside the central city of the metropolitan area. Information on metropolitan status was added by the Census Bureau, rather than being directly collected from respondents.
Concept:	Geographic Variables -- HOUSEHOLD
Start Position:	40
End Position:	40
Width:	1
Variable Format:	numeric
Implied Decimal Places:	0

Categories

Value	Label
0	Not identifiable
1	Not in metro area
2	Central city
3	Outside central city
4	Central city status unknown
9	Missing/Unknown

Variable: "COUNTY"

Name:	COUNTY
Label:	FIPS county code
Variable Text:	COUNTY gives the FIPS state and county codes for the respondent's county of residence. To preserve respondent confidentiality, not all counties are identified; however, about 45 percent of households in recent years are located in a county that is identified.
Concept:	Geographic Variables -- HOUSEHOLD
Start Position:	41
End Position:	45
Width:	5
Variable Format:	numeric
Implied Decimal Places:	0
Coder Instructions:	COUNTY is a five-digit numeric variable. The first two digits give the FIPS state code; the last three digits give the FIPS county code. For a list of counties identified in each state, follow the following links for the appropriate years:<br><br>September, 1995-April, 2004 [URL omitted from DDI.]<br>May, 2004-July,2005 [URL omitted from DDI.]<br>August, 2005 onward [URL omitted from DDI.]<br><br>00000 = Not identified

Variable: "METFIPS"

Name:	METFIPS
Label:	Metropolitan area FIPS code
Variable Text:	METFIPS gives the original (unrecoded) codes for the respondent's metropolitan area of residence. For the same variable recoded to FIPS codes from the 1990 census, see (METAREA) (definitions of metropolitan areas can change over time, however). METFIPS information was added to the ASEC CPS data by the Census Bureau, not collected from respondents.<br><br>Not all metropolitan areas are identified: see under "Codes" for more information. Note also that some component counties are not included in the CPS sample of households in certain metropolitan areas. See the "Specific Metropolitan Identifiers" Appendix of the appropriate month's technical documentation [URL omitted from

	DDI.] for more information on whether a specific metropolitan area sample has excluded components. For more information on the definitions and components of metropolitan areas over time, see the Census Bureau website [URL omitted from DDI.]; for the current metropolitan area definitions, see here [URL omitted from DDI.].<br><br>Note that the Census Bureau warns: "One set of estimates that can be produced from CPS microdata files should be treated with caution. These are estimates for individual metropolitan areas. Although estimates for the larger areas such as New York, Los Angeles, and so forth, should be fairly accurate and valid for a multitude of uses, estimates for the smaller metropolitan areas (those with populations under 500,000) should be used with caution because of the relatively large sampling variability associated with these estimates."
Concept:	Geographic Variables -- HOUSEHOLD
Start Position:	46
End Position:	50
Width:	5
Variable Format:	numeric
Implied Decimal Places:	0
Coder Instructions:	For a list of metropolitan areas identified and the corresponding codes, consult the links below for the appropriate time period. Note that some metropolitan areas are not available in all months of a time period.<br><br>1962 (ASEC) [URL omitted from DDI.]<br>1963-1967 (ASEC) [URL omitted from DDI.]<br>1968-1972 (ASEC) [URL omitted from DDI.]<br>1973-1975 (ASEC) [URL omitted from DDI.]<br>1976 (ASEC) [URL omitted from DDI.]<br>1977-1985 (ASEC) [URL omitted from DDI.]<br>1986-1987 (ASEC) [URL omitted from DDI.]<br>1988-March, 1995 [URL omitted from DDI.]<br>September, 1995-April, 2004 [URL omitted from DDI.]<br>May, 2004 -April, 2014 [URL omitted from DDI.]<br>May, 2014 onward [URL omitted from DDI.]<br><br>99998 = Unidentified or nonmetropolitan<br>99999 = Missing data

Variable: "CPI99"

Name:	CPI99

Label:	CPI-U adjustment factor to 1999 dollars
Variable Text:	CPI99 provides the CPI-U multiplier (available from the Bureau of Labor Statistics [URL omitted from DDI.]) to convert dollar figures to constant 1999 dollars. (This corresponds to the dollar amounts in the 2000 CPS, which inquired about income in 1999.) Multiplying dollar amounts by this variable (which is constant within years) will render them comparable across time and thus suitable for multivariate analysis.<br><br>For more information on how to use CPI99, please see the IPUMS inflation adjustment page [URL omitted from DDI.].
Concept:	Technical Variables -- HOUSEHOLD
Start Position:	51
End Position:	54
Width:	4
Variable Format:	numeric
Implied Decimal Places:	3
Coder Instructions:	CPI99 is a 4-digit variable with three implied decimal places.

Variable: "MONTH"

Name:	MONTH
Label:	Month
Variable Text:	MONTH indicates the calendar month of the CPS interview.
Concept:	Technical Variables -- HOUSEHOLD
Start Position:	55
End Position:	56
Width:	2
Variable Format:	numeric
Implied Decimal Places:	0

Categories

Value	Label
01	January
02	February
03	March
04	April
05	May
06	June
07	July
08	August
09	September
10	October
11	November
12	December

Variable: "PERNUM"

Name:	PERNUM
Label:	Person number in sample unit
Variable Text:	PERNUM numbers all persons within each household consecutively (starting with "1") in the order in which they are listed in the original CPS data. When combined with YEAR , MONTH, and SERIAL, PERNUM uniquely identifies each person within IPUMS-CPS samples, though not across IPUMS-CPS samples.
Concept:	Technical Variables -- PERSON
Start Position:	57
End Position:	58
Width:	2

Variable Format:	numeric
Implied Decimal Places:	0
Coder Instructions:	PERNUM is a 2-digit numeric variable.

Variable: "CPSIDP"

Name:	CPSIDP
Label:	CPSID, person record
Variable Text:	CPSIDP is an IPUMS-CPS defined variable that uniquely identifies individuals across CPS samples. The first six digits of CPSIDP index the four-digit year and two-digit month that the household was first in the CPS. CPSIDP allows users to link a respondent appearing with a designated household roster line number (LINENO) across samples, based on the 4-8-4 rotation pattern, by assigning a unique CPSIDP value to this line number. CPSIDP will only ever appear for a maximum of 8 times, which is the number of times a household may be observed in the CPS survey (as indexed by MISH). In some cases, individuals will appear fewer than 8 times due to migration, mortality, non-response, and recording errors. Extensive documentation about the creation of CPSIDP is available elsewhere [URL omitted from DDI.].<br><br>Users should note that it is important to verify CPSIDP linkages with AGE, SEX, and RACE. In some cases CPSIDP will result in erroneous links, which are due to errors in the source data. Cases with the same CPSIDP value may also have inconsistent responses across samples due to errors on the part of the respondent or in recording the response. Ultimately, it is up to the individual researcher to determine the acceptability of the linkages made using CPSIDP.<br><br>CPSIDP may also be used to link ASEC respondents who are in the March Basic Monthly file to other months of CPS data. This linking is made possible by IPUMS through the creation of MARBASECIDP.<br><br>To get started using CPSIDP, users may want to sort their data file by CPSIDP and MISH to create a person-time file.<br><br>Users should take care when including the March Basic or ASEC as part of their linking. Respondents who are part of the ASEC oversample (as indicated by ASECOVERP) have a CPSIDP value of 0. For further information about the relationship between the March Basic and the ASEC, please see our additional documentation [URL omitted from DDI.].
Concept:	Linking Variables -- PERSON
Start Position:	59
End Position:	72

Width:	14
Variable Format:	numeric
Implied Decimal Places:	0
Coder Instructions:	CPSIDP is a 14-digit numeric variable.

Variable: "WTSUPP"

Name:	WTSUPP
Label:	Supplement Weight
Variable Text:	WTSUPP is a person-level weight that should be used in analyses of individual-level CPS supplement data. Since the CPS relies on a complex stratified sampling scheme, it is essential to use one of the provided weighting variables.<br><br>Researchers should use WTFINL rather than WTSUPP when they wish to conduct person-level analyses of non-supplement data. EARNWT should be used for any analysis including a small number of person-level variables (EARNWEEK, HOURWAGE, PAIDHOUR, and UNION). Researchers should use HWTSUPP for household-level analyses.<br><br>User Caution: For analyses that include the 2014 ASEC sample, please see the comparability tab.<br><br>The ASEC CPS files include two groups of people who are not included in the production of published labor force statistics: (1) members of the armed services, and (2) members of the Hispanic oversample who were interviewed in months other than March. WTFINL and EARNWT assign these groups a value of 0. Both groups are assigned non-zero values in WTSUPP.<br><br>WTSUPP is based on the inverse probability of selection into the sample and adjustments for the following factors: failure to obtain an interview; sampling within large sample units; the known distribution of the entire population according to age, sex, and race; over-sampling Hispanic persons; to give husbands and wives the same weight; and an additional step to provide consistency with labor force estimates from the basic survey.<br><br>Estimates on the entire population are prepared by projecting forward the resident population from the last available census. These projections are derived by updating the demographic census data from a number of other data sources that account for death, births and net migration. About 3 years after every census (i.e. 2003 for the 2000 Census and 2013 for the 2010 Census), the Census Bureau updates its independent population control and provides a new weight for the relevant years.<br><br>Two important points should be noted here. First, the lag between when the Census is conducted and when the CPS weights are updated is about 3 years. While the Census data are being processed, the CPS files are made available using the weighting scheme from the US Census prior to the latest Census. Second, once the files are updated, the old weights become obsolete and are replaced in the IPUMS data extract system.

	Published estimates from the lag years that use the old weights are not always updated. For example, 2010 poverty estimates were released in ASEC using the 2000 population controls. Once the 2010 population controls were made available, IPUMS-CPS replaced the ASEC 2010, 2011, and 2012 weights that are based on the 2000 population control with weights that are based on the 2010 population controls. IPUMS-CPS makes available only the most up-to-date weights.
Concept:	Technical Variables -- PERSON
Start Position:	73
End Position:	82
Width:	10
Variable Format:	numeric
Implied Decimal Places:	4
Coder Instructions:	WTSUPP is a 10-digit numeric variable with four implied decimal places. That is, values of 0012345600 should be interpreted as 1,234.56. The IPUMS command files automatically divide WTSUPP by 10,000, so no further adjustment is needed.

Variable: "EARNWT"

Name:	EARNWT
Label:	Earnings weight
Variable Text:	EARNWT is a person-level weight that should be used in any analysis including one of the following variables: EARNWEEK, HOURWAGE, PAIDHOUR, UNION, UHRSWORKORG, WKSWORKORG, ELIGORG, and OTPAY. For any other analysis using ASEC data, researchers should use WTSUPP or for analyses of non-ASEC data, WTFINL.<br><br>Individuals in the 6 rotation groups that were not asked the "earner study" questions (covering EARNWEEK, HOURWAGE, PAIDHOUR, UNION, UHRSWORKORG, WKSWORKORG, ELIGORG, and OTPAY) have a value of zero for EARNWT. Even in the 2 rotation groups where "earner study" questions were fielded, children under 15 and members of the armed forces have a value of zero for EARNWT.<br><br>According to Technical Paper 66 [URL omitted from DDI.], issued jointly by the Census Bureau and the Bureau of Labor Statistics, individuals eligible for the earner study are civilians age 15 and older in rotation groups 4 or 8 who are not self-employed. In any given month, approximately 1/4 of the CPS sample is in the earner study and each household should appear in the earner study exactly twice. Based on documentation from Unicon and NBER [URL omitted from DDI.], and after an inspection of the original CPS data, we recommend that users impose the CPS eligibility restrictions in any analyses of earner study variables.

Concept:	Technical Variables -- PERSON
Start Position:	83
End Position:	92
Width:	10
Variable Format:	numeric
Implied Decimal Places:	4
Coder Instructions:	EARNWT is an 8-digit numeric variable with four implied decimals. That is, 12345678 should be interpreted as 1234.5678. The IPUMS command files automatically divide EARNWT by 10000, so no further adjustment is needed.

Variable: "NCHILD"

Name:	NCHILD
Label:	Number of own children in household
Variable Text:	NCHILD counts the number of own children (of any age or marital status) residing with each individual. NCHILD includes step-children and adopted children as well as biological children. Persons with no children present are coded 0.<br><br>Note that NCHILD is an IPUMS-derived variable using IPUMS-derived family interrelationships. Thus NCHILD may differ from any family information that comes from just the Census family definitions. See for example FTYPE, FAMKIND, and FAMREL for more on Census family units.
Concept:	Family Interrelationship Variables -- PERSON
Start Position:	93
End Position:	93
Width:	1
Variable Format:	numeric
Implied Decimal	0

Places:	

Categories

Value	Label
0	0 children present
1	1 child present
2	2
3	3
4	4
5	5
6	6
7	7
8	8
9	9+

Variable: "AGE"

Name:	AGE
Label:	Age
Variable Text:	Age gives each person's age at last birthday.
Concept:	Core Demographic Variables -- PERSON
Start Position:	94
End Position:	95
Width:	2
Variable Format:	numeric
Implied Decimal Places:	0

Categories

Value	Label
00	Under 1 year
01	1
02	2
03	3
04	4
05	5
06	6
07	7
08	8
09	9
10	10
11	11
12	12
13	13
14	14
15	15
16	16
17	17
18	18
19	19
20	20

21	21
22	22
23	23
24	24
25	25
26	26
27	27
28	28
29	29
30	30
31	31
32	32
33	33
34	34
35	35
36	36
37	37
38	38
39	39
40	40
41	41
42	42
43	43
44	44

45	45
46	46
47	47
48	48
49	49
50	50
51	51
52	52
53	53
54	54
55	55
56	56
57	57
58	58
59	59
60	60
61	61
62	62
63	63
64	64
65	65
66	66
67	67
68	68

69	69
70	70
71	71
72	72
73	73
74	74
75	75
76	76
77	77
78	78
79	79
80	80
81	81
82	82
83	83
84	84
85	85
86	86
87	87
88	88
89	89
90	90 (90+, 1988-2002)
91	91
92	92

93	93
94	94
95	95
96	96
97	97
98	98
99	99+

Variable: "SEX"

Name:	SEX
Label:	Sex
Variable Text:	SEX gives each person's sex.
Concept:	Core Demographic Variables -- PERSON
Start Position:	96
End Position:	96
Width:	1
Variable Format:	numeric
Implied Decimal Places:	0

Categories

Value	Label
1	Male
2	Female
9	NIU

Variable: "RACE"

Name:	RACE
Label:	Race
Variable Text:	Racial categories in the CPS have been more consistent than racial categories in the census. Up through 2002, the number of race categories ranged from 3 (white, negro, and other) to 5 (white, black, American Indian/Eskimo/Aleut, Asian or Pacific Islander, and other). Beginning in 2003, respondents could report more than one race, and the number of codes rose to 21, and then up to 26 codes in 2013.
Concept:	Core Demographic Variables -- PERSON
Start Position:	97
End Position:	99
Width:	3
Variable Format:	numeric
Implied Decimal Places:	0

Categories

Value	Label
100	White
200	Black/Negro
300	American Indian/Aleut/Eskimo
650	Asian or Pacific Islander
651	Asian only
652	Hawaiian/Pacific Islander only
700	Other (single) race, n.e.c.
801	White-Black

802	White-American Indian
803	White-Asian
804	White-Hawaiian/Pacific Islander
805	Black-American Indian
806	Black-Asian
807	Black-Hawaiian/Pacific Islander
808	American Indian-Asian
809	Asian-Hawaiian/Pacific Islander
810	White-Black-American Indian
811	White-Black-Asian
812	White-American Indian-Asian
813	White-Asian-Hawaiian/Pacific Islander
814	White-Black-American Indian-Asian
815	American Indian-Hawaiian/Pacific Islander
816	White-Black--Hawaiian/Pacific Islander
817	White-American Indian-Hawaiian/Pacific Islander
818	Black-American Indian-Asian
819	White-American Indian-Asian-Hawaiian/Pacific Islander
820	Two or three races, unspecified
830	Four or five races, unspecified
999	Blank

Variable: "MARST"

Name:	MARST

Label:	Marital status
Variable Text:	MARST gives each person's current marital status, including whether the spouse was currently living in the same household.
Concept:	Core Demographic Variables -- PERSON
Start Position:	100
End Position:	100
Width:	1
Variable Format:	numeric
Implied Decimal Places:	0

Categories

Value	Label
1	Married, spouse present
2	Married, spouse absent
3	Separated
4	Divorced
5	Widowed
6	Never married/single
7	Widowed or Divorced
9	NIU

Variable: "POPSTAT"

Name:	POPSTAT
Label:	Adult civilian, armed forces, or child
Variable	POPSTAT reports the person's status in the population -- whether the person is an adult

Text:	civilian, member of the U. S. armed forces, or a child.<br><br>The CPS is, in large part, a labor market survey, and is used to measure unemployment among the civilian labor force. (The U.S. unemployment rate reported by the Bureau of Labor Statistics excludes members of the armed forces.) Children (for ASEC samples under age 14 through 1979 and under age 15 beginning in 1980; for non-ASEC samples under 14 through February, 1989 and under 15 beginning March 1989) were not asked questions pertaining to economic activity. Members of the armed forces were asked only a small number of questions relating to demographic facts, migration, and income during the previous calendar year. POPSTAT provides a useful "filter" variable for excluding persons who had no responses for many of the survey questions. If children and/or members of the armed forces were excluded from the universe of a particular question, they appear only in the "not in universe" category of a variable.
Concept:	Core Demographic Variables -- PERSON
Start Position:	101
End Position:	101
Width:	1
Variable Format:	numeric
Implied Decimal Places:	0

Categories

Value	Label
1	Adult civilian
2	Armed Forces
3	Child

Variable: "BPL"

Name:	BPL
Label:	Birthplace
Variable Text:	BPL indicates whether persons were born in the United States and, if not, the foreign country where they were born.

Concept:	Ethnicity/Nativity Variables -- PERSON
Start Position:	102
End Position:	106
Width:	5
Variable Format:	numeric
Implied Decimal Places:	0

Categories

Value	Label
09900	United States, n.s.
10000	American Samoa
10500	Guam
10750	Northern Mariana Islands
11000	Puerto Rico
11500	U.S. Virgin Islands
12090	U.S. outlying areas, n.s.
15000	Canada
16010	Bermuda
19900	North America, n.s.
20000	Mexico
21010	Belize/British Honduras
21020	Costa Rica
21030	El Salvador
21040	Guatemala

21050	Honduras
21060	Nicaragua
21070	Panama
21090	Central America, n.s.
25000	Cuba
26010	Dominican Republic
26020	Haiti
26030	Jamaica
26043	Bahamas
26044	Barbados
26054	Dominica
26055	Grenada
26060	Trinidad and Tobago
26065	Antigua and Barbuda
26070	St. Kitts--Nevis
26075	St. Lucia
26080	St. Vincent and the Grenadi
26091	Caribbean, n.s.
30005	Argentina
30010	Bolivia
30015	Brazil
30020	Chile
30025	Colombia
30030	Ecuador

30040	Guyana/British Guiana
30050	Peru
30060	Uruguay
30065	Venezuela
30070	Paraguay
30090	South America, n.s.
31000	Americas, n.s.
40000	Denmark
40100	Finland
40200	Iceland
40400	Norway
40500	Sweden
41000	England
41100	Scotland
41200	Wales
41300	United Kingdom, n.s.
41400	Ireland
41410	Northern Ireland
42000	Belgium
42100	France
42500	Netherlands
42600	Switzerland
43300	Greece
43400	Italy

43600	Portugal
43610	Azores
43800	Spain
45000	Austria
45200	Czechoslavakia
45212	Slovakia
45213	Czech Republic
45300	Germany
45400	Hungary
45500	Poland
45600	Romania
45650	Bulgaria
45675	Albania
45700	Yugoslavia
45720	Bosnia and Herzegovina
45730	Croatia
45740	Macedonia
45750	Serbia
45760	Kosovo
45770	Montenego
46100	Estonia
46200	Latvia
46300	Lithuania
46500	Other USSR/Russia

46530	Ukraine
46535	Belarus
46540	Moldova
46590	USSR, n.s.
49900	Europe, n.s.
50000	China
50010	Hong Kong
50040	Taiwan
50100	Japan
50200	Korea
50220	South Korea
50300	Mongolia
51100	Cambodia
51200	Indonesia
51300	Laos
51400	Malaysia
51500	Philippines
51600	Singapore
51700	Thailand
51800	Vietnam
52000	Afghanistan
52100	India
52110	Bangladesh
52120	Bhutan

52130	Burma
52140	Pakistan
52150	Sri Lanka
52200	Nepal
55100	Armenia
55200	Azerbaijan
55300	Georgia
55400	Uzbekistan
55500	Kazakhstan
53000	Iran
53200	Iraq
53400	Israel
53420	Palestine
53500	Jordan
53700	Lebanon
54000	Saudi Arabia
54100	Syria
54200	Turkey
54300	Cyprus
54350	Kuwait
54400	Yemen
54500	United Arab Emirates
54700	Middle East, n.s.
59900	Asia, n.e.c./n.s.

60010	Northern Africa
60012	Egypt/United Arab Rep.
60014	Morocco
60016	Algeria
60018	Sudan
60019	Libya
60023	Ghana
60031	Nigeria
60032	Cameroon
60033	Cape Verde
60034	Liberia
60035	Senegal
60036	Sierra Leone
60037	Guinea
60038	Ivory Coast
60039	Togo
60040	Eritrea
60044	Ethiopia
60045	Kenya
60050	Somalia
60060	Tanzania
60065	Uganda
60070	Zimbabwe
60094	South Africa (Union of)

60095	Zaire
60096	Congo
60097	Zambia
60099	Africa, n.s./n.e.c.
70010	Australia
70020	New Zealand
71000	Pacific Islands
71021	Fiji
71022	Tonga
71023	Samoa
71024	Marshall Islands
72000	Micronesia
96000	Other, n.e.c. and unknown
99999	NIU

Variable: "YRIMMIG"

Name:	YRIMMIG
Label:	Year of immigration
Variable Text:	YRIMMIG reports the year in which a person born outside the United States "came to the U.S. to stay."
Concept:	Ethnicity/Nativity Variables -- PERSON
Start Position:	107
End Position:	110
Width:	4
Variable Format:	numeric

Implied Decimal Places:	0

Categories

Value	Label
0000	NIU
1949	1949 or earlier
1959	1950-1959
1964	1960-1964
1969	1965-1969
1974	1970-1974
1979	1975-1979
1981	1980-1981
1983	1982-1983
1985	1984-1985
1987	1986-1987
1989	1988-1989
1991	1990-1991
1993	1992-1993
1994	1992-1994
1995	1994-1995
1996	1994-1996
1997	1996-1997
1998	1996-1998 (2000 CPS: 1998)
1999	1998-1999 (1999 CPS: 1996-1999)

2000	1998-2000
2001	2000-2001 (2001 CPS: 1998-2001)
2002	2000-2002
2003	2002-2003 (2003 CPS: 2000-2003)
2004	2002-2004
2005	2004-2005 (2005 CPS: 2002-2005)
2006	2004-2006
2007	2004-2007
2008	2006-2008 (2006-2007 CPS: 2004-2008)
2009	2006-2009
2010	2008-2010 (2012 CPS: 2008-2009)
2011	2008-2011
2012	2010-2012 (2014 CPS: 2010-2011)
2013	2010-2013
2014	2012-2014
2015	2012-2015
2016	2014-2016

Variable: "CITIZEN"

Name:	CITIZEN
Label:	Citizenship status
Variable Text:	CITIZEN reports the citizenship status of foreign-born persons. In IPUMS-CPS, people born in the U.S., Puerto Rico, or U.S. outlying areas were excluded from the question universe. Respondents were identified as belonging to one of three groups: citizens by virtue of being born abroad to American parents; naturalized citizens; and non-citizens.
Concept:	Ethnicity/Nativity Variables -- PERSON

Start Position:	111
End Position:	111
Width:	1
Variable Format:	numeric
Implied Decimal Places:	0

Categories

Value	Label
0	NIU
1	Born abroad of American parents
2	Naturalized citizen
3	Not a citizen
9	NIU

Variable: "NATIVITY"

Name:	NATIVITY
Label:	Foreign birthplace or parentage
Variable Text:	NATIVITY classifies each person as native-born or foreign-born (i.e., whether a first-generation immigrant) and further specifies whether the parents of a native-born person were native-born or foreign-born (i.e., whether a second-generation immigrant). NATIVITY is constructed from information in the BPL, FBPL, and MBPL variables, which respectively report the place of birth of the respondent and his or her father and mother. Persons born in outlying U.S. territories and possessions and those born abroad to U.S. parents are treated as foreign-born in NATIVITY.
Concept:	Ethnicity/Nativity Variables -- PERSON
Start Position:	112

End Position:	112
Width:	1
Variable Format:	numeric
Implied Decimal Places:	0

Categories

Value	Label
0	Unknown
1	Both parents native-born
2	Father foreign, mother native
3	Mother foreign, father native
4	Both parents foreign
5	Foreign born

Variable: "HISPAN"

Name:	HISPAN
Label:	Hispanic origin
Variable Text:	HISPAN identifies and classifies persons of Hispanic/Spanish/Latino origin. Origin is ancestry, lineage, heritage, national group, or country of birth.<br><br>Prior to 2003, information was collected by asking, "What is the origin or descent of each person in this household?" and asking the respondent to select the appropriate category from a limited number of choices on a flashcard (including "another group not listed.") The choices included five to eight choices that would be classified as Hispanic, "Negro" and "Black," and a small number of European ancestry groups such as "German."<br><br>The primary intention of the question was to identify Hispanic respondents, rather than origin or descent for the general population. Beginning in 1976, the original CPS data preserved detail for only the Hispanic responses, with all others answers lumped together as "another group not listed" (relabeled "Not Hispanic" in IPUMS-CPS).<br><br>In 2003 and later years, respondents were asked, "Are you Spanish, Hispanic, or

	Latino?" rather than the broad query about origin or descent. Detailed information about Hispanic ethnicity was collected only from those who answered "yes" to this initial question.
Concept:	Ethnicity/Nativity Variables -- PERSON
Start Position:	113
End Position:	115
Width:	3
Variable Format:	numeric
Implied Decimal Places:	0

Categories

Value	Label
000	Not Hispanic
100	Mexican
102	Mexican American
103	Mexicano/Mexicana
104	Chicano/Chicana
108	Mexican (Mexicano)
109	Mexicano/Chicano
200	Puerto Rican
300	Cuban
400	Dominican
500	Salvadoran

401	Other Hispanic
410	Central/South American
411	Central American, (excluding Salvadoran)
412	South American
901	Do not know
902	N/A (and no response 1985-87)

Variable: "EDUC"

Name:	EDUC
Label:	Educational attainment recode
Variable Text:	EDUC indicates respondents' educational attainment, as measured by the highest year of school or degree completed. Note that completion differs from the highest year of school attendance; for example, respondents who attended 10th grade but did not finish were classified in EDUC as having completed 9th grade.<br><br>EDUC is a combination of two other variables, HIGRADE and EDUC99, which measure educational attainment in different ways. HIGRADE is available for years prior to 1992 and gives the respondent's highest grade of school or year of college completed. EDUC99 is available beginning in 1992 and classifies high school graduates according to their highest degree or diploma attained.<br><br>General and detailed codes are not yet available for IPUMS-CPS, but one can construct the general version of EDUC by reading only the first two columns of EDUC.
Concept:	Education Variables -- PERSON
Start Position:	116
End Position:	118
Width:	3
Variable Format:	numeric
Implied Decimal Places:	0

Categories

Value	Label
000	NIU or no schooling
001	NIU or blank
002	None or preschool
010	Grades 1, 2, 3, or 4
011	Grade 1
012	Grade 2
013	Grade 3
014	Grade 4
020	Grades 5 or 6
021	Grade 5
022	Grade 6
030	Grades 7 or 8
031	Grade 7
032	Grade 8
040	Grade 9
050	Grade 10
060	Grade 11
070	Grade 12
071	12th grade, no diploma
072	12th grade, diploma unclear
073	High school diploma or equivalent

080	1 year of college
081	Some college but no degree
090	2 years of college
091	Associate's degree, occupational/vocational program
092	Associate's degree, academic program
100	3 years of college
110	4 years of college
111	Bachelor's degree
120	5+ years of college
121	5 years of college
122	6+ years of college
123	Master's degree
124	Professional school degree
125	Doctorate degree
999	Missing/Unknown

Variable: "EDUC99"

Name:	EDUC99
Label:	Educational attainment, 1990
Variable Text:	EDUC99 reports the respondent's highest level of educational attainment. Respondents without high school diplomas were to indicate the highest school grade they had completed, while those with high school diplomas were to indicate the highest diploma or degree they had obtained.
Concept:	Education Variables -- PERSON
Start Position:	119
End Position:	120

Width:	2
Variable Format:	numeric
Implied Decimal Places:	0

Categories

Value	Label
00	NIU
01	No school completed
04	1st-4th grade
05	5th-8th grade
06	9th grade
07	10th grade
08	11th grade
09	12th grade, no diploma
10	High school graduate, or GED
11	Some college, no degree
12	Associate degree, type of program not specified
13	Associate degree, occupational program
14	Associate degree, academic program
15	Bachelors degree
16	Masters degree
17	Professional degree
18	Doctorate degree

Variable: "EMPSTAT"

Name:	EMPSTAT
Label:	Employment status
Variable Text:	EMPSTAT indicates whether persons were part of the labor force--working or seeking work--and, if so, whether they were currently unemployed. The variable also provides information on the activity (e.g., doing housework, attending school,) or status (e.g., retired, unable to work) of persons not in the labor force, as well as limited additional information on those who are in the labor force (e.g. members of the Armed Forces, those with a job, but not at work last week). See LABFORCE for a dichotomous variable identifying whether a person participated in the labor force.<br><br>In the CPS, individuals' employment status was determined on the basis of answers to a series of questions relating to their activities during the preceding week. Those who reported doing any work at all for pay or profit, or working at least fifteen hours without pay in a family business or farm, were classified as "at work." Those who did not work during the previous week but who acknowledged having a job or business from which they were temporarily absent (e.g., due to illness, vacation, bad weather, or labor dispute) were also classified as employed, under the heading "has job, not at work last week."<br><br>Because the CPS is designed to measure unemployment in the civilian population, the original employment status variable in the survey classifies members of the armed forces as NIU (Not in universe).<br><br>Unemployed persons make up the third element of the labor force. Individuals were coded as unemployed if they did no work for pay or profit, did not have a job from which they were briefly absent, and either reported looking for work as their major activity during the previous week (for 1962 through 1993) or answered yes to a question about whether they had been looking for work in the past four weeks. People who were temporarily laid off from a job were also classified as unemployed. A separate CPS variable specifying whether an unemployed person had worked before or was looking for a first job was used to distinguish between "experienced" and "inexperienced" unemployed persons in IPUMS-CPS.<br><br>Persons who were neither employed nor unemployed fall into the residual category, "not in labor force." Such individuals might be retired, disabled due to an illness lasting at least 6 months, occupied with other activities such as attending school or keeping house, or convinced that they are unlikely to find employment (discouraged workers).
Concept:	Work Variables -- PERSON
Start Position:	121
End Position:	122
Width:	2
Variable Format:	numeric

Implied Decimal Places:	0

Categories

Value	Label
00	NIU
01	Armed Forces
10	At work
12	Has job, not at work last week
20	Unemployed
21	Unemployed, experienced worker
22	Unemployed, new worker
30	Not in labor force
31	NILF, housework
32	NILF, unable to work
33	NILF, school
34	NILF, other
35	NILF, unpaid, lt 15 hours
36	NILF, retired

Variable: "OCC"

Name:	OCC
Label:	Occupation
Variable Text:	OCC reports the person's primary occupation. Respondents who held more than one job were to report the job at which they worked the largest number of hours. For persons who were employed at the time of the survey, OCC relates to the job worked during the preceding week; unemployed persons and those not currently in the labor force were to give their most recent occupation. The CPS interviewer collected

	information by asking what kind of work the person was doing, and Census Bureau staff coded the information into the contemporary CPS or census occupational classification. Researchers who wish to work with a consistent occupational coding scheme for 1968 forward should use the OCC1950 variable. For general discussion of employment concepts, including the definition of those not in the labor force, see the documentation on EMPSTAT.
Concept:	Work Variables -- PERSON
Start Position:	123
End Position:	126
Width:	4
Variable Format:	numeric
Implied Decimal Places:	0
Coder Instructions:	OCC is a 4-digit numeric variable.<br>(Codes for 1962-1967 are 2 digits; each is preceded by two zeros).<br>(Codes for 1968-2002 are 3 digits; each is preceded by a zero in the first position.)<br><br>1962-1967 [URL omitted from DDI.]<br>1968-1970 [URL omitted from DDI.]<br>1971-1982 [URL omitted from DDI.]<br>1983-1991 [URL omitted from DDI.]<br>1992-2002 [URL omitted from DDI.]<br>2003-2010 [URL omitted from DDI.]<br>2011+ [URL omitted from DDI.]

Variable: "OCC2010"

Name:	OCC2010
Label:	Occupation, 2010 basis
Variable Text:	OCC2010 is a harmonized occupation coding scheme based on the Census Bureau's 2010 occupation classification scheme. Similar variables are offered for the 1950 (OCC1950) and 1990 (OCC1990) classification codes. OCC2010 offers researchers a consistent, long-term classification of occupations.<br><br>The occupational coding scheme in CPS data has changed several times since the 1960s. The CPS's occupational coding scheme has mirrored that of the Census Bureau, though the CPS has always introduced major coding changes one-to-three years later than the Census Bureau. All original occupational information is stored in the OCC variable. The meaning of codes in the OCC variable changes with each new coding scheme.<br><br>In the interest of harmonization, however, the scheme has been modified to achieve the

	most consistent categories across time. That is, some categories that provide more detail in the 2010 scheme were grouped together because earlier categories are inseparable when more than one occupation is coded together. For users who wish to further aggregate occupation to broader categories, the 2010 scheme is generally organized by the following groups:<br><br>Management in Business, Science, and Arts = 10-430<br>Business Operations Specialists = 500-730<br>Financial Specialists = 800-950<br>Computer and Mathematical = 1000-1240<br>Architecture and Engineering = 1300-1540<br>Technicians = 1550-1560<br>Life, Physical, and Social Science = 1600-1980<br>Community and Social Services = 2000-2060<br>Legal = 2100-2150<br>Education, Training, and Library = 2200-2550<br>Arts, Design, Entertainment, Sports, and Media = 2600-2920<br>Healthcare Practitioners and Technicians = 3000-3540<br>Healthcare Support = 3600-3650<br>Protective Service = 3700-3950<br>Food Preparation and Serving = 4000-4150<br>Building and Grounds Cleaning and Maintenance = 4200-4250<br>Personal Care and Service = 4300-4650<br>Sales and Related = 4700-4965<br>Office and Administrative Support = 5000-5940<br>Farming, Fisheries, and Forestry = 6005-6130<br>Construction = 6200-6765<br>Extraction = 6800-6940<br>Installation, Maintenance, and Repair = 7000-7630<br>Production = 7700-8965<br>Transportation and Material Moving = 9000-9750<br>Military = 9800-9830<br>No Occupation = 9920<br><br>We followed a process of constructing and testing OCC2010 that is similar to OCC1990's process, which is discussed in more detail in this BLS working paper [URL omitted from DDI.]. We performed a variety of tests to ensure that the new categories are as robust as possible over the long-term. Please also see the description tab for OCC1990 for further detail about our process.
Concept:	Work Variables -- PERSON
Start Position:	127
End Position:	130
Width:	4
Variable Format:	numeric
Implied Decimal Places:	0

Categories

Value	Label
0010	Chief executives and legislators/public administration
0020	General and Operations Managers
0030	Managers in Marketing, Advertising, and Public Relations
0100	Administrative Services Managers
0110	Computer and Information Systems Managers
0120	Financial Managers
0130	Human Resources Managers
0140	Industrial Production Managers
0150	Purchasing Managers
0160	Transportation, Storage, and Distribution Managers
0205	Farmers, Ranchers, and Other Agricultural Managers
0220	Constructions Managers
0230	Education Administrators
0300	Architectural and Engineering Managers
0310	Food Service and Lodging Managers
0320	Funeral Directors
0330	Gaming Managers
0350	Medical and Health Services Managers
0360	Natural Science Managers
0410	Property, Real Estate, and Community Association Managers
0420	Social and Community Service Managers

0430	Managers, nec (including Postmasters)
0500	Agents and Business Managers of Artists, Performers, and Athletes
0510	Buyers and Purchasing Agents, Farm Products
0520	Wholesale and Retail Buyers, Except Farm Products
0530	Purchasing Agents, Except Wholesale, Retail, and Farm Products
0540	Claims Adjusters, Appraisers, Examiners, and Investigators
0560	Compliance Officers, Except Agriculture
0600	Cost Estimators
0620	Human Resources, Training, and Labor Relations Specialists
0700	Logisticians
0710	Management Analysts
0720	Meeting and Convention Planners
0730	Other Business Operations and Management Specialists
0800	Accountants and Auditors
0810	Appraisers and Assessors of Real Estate
0820	Budget Analysts
0830	Credit Analysts
0840	Financial Analysts
0850	Personal Financial Advisors
0860	Insurance Underwriters
0900	Financial Examiners
0910	Credit Counselors and Loan Officers
0930	Tax Examiners and Collectors, and Revenue Agents
0940	Tax Preparers

0950	Financial Specialists, nec
1000	Computer Scientists and Systems Analysts/Network systems Analysts/Web Developers
1010	Computer Programmers
1020	Software Developers, Applications and Systems Software
1050	Computer Support Specialists
1060	Database Administrators
1100	Network and Computer Systems Administrators
1200	Actuaries
1220	Operations Research Analysts
1230	Statisticians
1240	Mathematical science occupations, nec
1300	Architects, Except Naval
1310	Surveyors, Cartographers, and Photogrammetrists
1320	Aerospace Engineers
1350	Chemical Engineers
1360	Civil Engineers
1400	Computer Hardware Engineers
1410	Electrical and Electronics Engineers
1420	Environmental Engineers
1430	Industrial Engineers, including Health and Safety
1440	Marine Engineers and Naval Architects
1450	Materials Engineers
1460	Mechanical Engineers
1520	Petroleum, mining and geological engineers, including mining safety engineers

1530	Engineers, nec
1540	Drafters
1550	Engineering Technicians, Except Drafters
1560	Surveying and Mapping Technicians
1600	Agricultural and Food Scientists
1610	Biological Scientists
1640	Conservation Scientists and Foresters
1650	Medical Scientists, and Life Scientists, All Other
1700	Astronomers and Physicists
1710	Atmospheric and Space Scientists
1720	Chemists and Materials Scientists
1740	Environmental Scientists and Geoscientists
1760	Physical Scientists, nec
1800	Economists and market researchers
1820	Psychologists
1830	Urban and Regional Planners
1840	Social Scientists, nec
1900	Agricultural and Food Science Technicians
1910	Biological Technicians
1920	Chemical Technicians
1930	Geological and Petroleum Technicians, and Nuclear Technicians
1960	Life, Physical, and Social Science Technicians, nec
1980	Professional, Research, or Technical Workers, nec
2000	Counselors

2010	Social Workers
2020	Community and Social Service Specialists, nec
2040	Clergy
2050	Directors, Religious Activities and Education
2060	Religious Workers, nec
2100	Lawyers, and judges, magistrates, and other judicial workers
2140	Paralegals and Legal Assistants
2150	Legal Support Workers, nec
2200	Postsecondary Teachers
2300	Preschool and Kindergarten Teachers
2310	Elementary and Middle School Teachers
2320	Secondary School Teachers
2330	Special Education Teachers
2340	Other Teachers and Instructors
2400	Archivists, Curators, and Museum Technicians
2430	Librarians
2440	Library Technicians
2540	Teacher Assistants
2550	Education, Training, and Library Workers, nec
2600	Artists and Related Workers
2630	Designers
2700	Actors, Producers, and Directors
2720	Athletes, Coaches, Umpires, and Related Workers
2740	Dancers and Choreographers

2750	Musicians, Singers, and Related Workers
2760	Entertainers and Performers, Sports and Related Workers, All Other
2800	Announcers
2810	Editors, News Analysts, Reporters, and Correspondents
2825	Public Relations Specialists
2840	Technical Writers
2850	Writers and Authors
2860	Media and Communication Workers, nec
2900	Broadcast and Sound Engineering Technicians and Radio Operators, and media and communication equipment workers, all other
2910	Photographers
2920	Television, Video, and Motion Picture Camera Operators and Editors
3000	Chiropractors
3010	Dentists
3030	Dieticians and Nutritionists
3040	Optometrists
3050	Pharmacists
3060	Physicians and Surgeons
3110	Physician Assistants
3120	Podiatrists
3130	Registered Nurses
3140	Audiologists
3150	Occupational Therapists
3160	Physical Therapists

3200	Radiation Therapists
3210	Recreational Therapists
3220	Respiratory Therapists
3230	Speech Language Pathologists
3240	Therapists, nec
3250	Veterinarians
3260	Health Diagnosing and Treating Practitioners, nec
3300	Clinical Laboratory Technologists and Technicians
3310	Dental Hygienists
3320	Diagnostic Related Technologists and Technicians
3400	Emergency Medical Technicians and Paramedics
3410	Health Diagnosing and Treating Practitioner Support Technicians
3500	Licensed Practical and Licensed Vocational Nurses
3510	Medical Records and Health Information Technicians
3520	Opticians, Dispensing
3530	Health Technologists and Technicians, nec
3540	Healthcare Practitioners and Technical Occupations, nec
3600	Nursing, Psychiatric, and Home Health Aides
3610	Occupational Therapy Assistants and Aides
3620	Physical Therapist Assistants and Aides
3630	Massage Therapists
3640	Dental Assistants
3650	Medical Assistants and Other Healthcare Support Occupations, nec
3700	First-Line Supervisors of Correctional Officers

3710	First-Line Supervisors of Police and Detectives
3720	First-Line Supervisors of Fire Fighting and Prevention Workers
3730	Supervisors, Protective Service Workers, All Other
3740	Firefighters
3750	Fire Inspectors
3800	Sheriffs, Bailiffs, Correctional Officers, and Jailers
3820	Police Officers and Detectives
3900	Animal Control
3910	Private Detectives and Investigators
3930	Security Guards and Gaming Surveillance Officers
3940	Crossing Guards
3950	Law enforcement workers, nec
4000	Chefs and Cooks
4010	First-Line Supervisors of Food Preparation and Serving Workers
4030	Food Preparation Workers
4040	Bartenders
4050	Combined Food Preparation and Serving Workers, Including Fast Food
4060	Counter Attendant, Cafeteria, Food Concession, and Coffee Shop
4110	Waiters and Waitresses
4120	Food Servers, Nonrestaurant
4130	Food preparation and serving related workers, nec
4140	Dishwashers
4150	Host and Hostesses, Restaurant, Lounge, and Coffee Shop
4200	First-Line Supervisors of Housekeeping and Janitorial Workers

4210	First-Line Supervisors of Landscaping, Lawn Service, and Groundskeeping Workers
4220	Janitors and Building Cleaners
4230	Maids and Housekeeping Cleaners
4240	Pest Control Workers
4250	Grounds Maintenance Workers
4300	First-Line Supervisors of Gaming Workers
4320	First-Line Supervisors of Personal Service Workers
4340	Animal Trainers
4350	Nonfarm Animal Caretakers
4400	Gaming Services Workers
4420	Ushers, Lobby Attendants, and Ticket Takers
4430	Entertainment Attendants and Related Workers, nec
4460	Funeral Service Workers and Embalmers
4500	Barbers
4510	Hairdressers, Hairstylists, and Cosmetologists
4520	Personal Appearance Workers, nec
4530	Baggage Porters, Bellhops, and Concierges
4540	Tour and Travel Guides
4600	Childcare Workers
4610	Personal Care Aides
4620	Recreation and Fitness Workers
4640	Residential Advisors
4650	Personal Care and Service Workers, All Other
4700	First-Line Supervisors of Sales Workers

4720	Cashiers
4740	Counter and Rental Clerks
4750	Parts Salespersons
4760	Retail Salespersons
4800	Advertising Sales Agents
4810	Insurance Sales Agents
4820	Securities, Commodities, and Financial Services Sales Agents
4830	Travel Agents
4840	Sales Representatives, Services, All Other
4850	Sales Representatives, Wholesale and Manufacturing
4900	Models, Demonstrators, and Product Promoters
4920	Real Estate Brokers and Sales Agents
4930	Sales Engineers
4940	Telemarketers
4950	Door-to-Door Sales Workers, News and Street Vendors, and Related Workers
4965	Sales and Related Workers, All Other
5000	First-Line Supervisors of Office and Administrative Support Workers
5010	Switchboard Operators, Including Answering Service
5020	Telephone Operators
5030	Communications Equipment Operators, All Other
5100	Bill and Account Collectors
5110	Billing and Posting Clerks
5120	Bookkeeping, Accounting, and Auditing Clerks
5130	Gaming Cage Workers

5140	Payroll and Timekeeping Clerks
5150	Procurement Clerks
5160	Bank Tellers
5165	Financial Clerks, nec
5200	Brokerage Clerks
5220	Court, Municipal, and License Clerks
5230	Credit Authorizers, Checkers, and Clerks
5240	Customer Service Representatives
5250	Eligibility Interviewers, Government Programs
5260	File Clerks
5300	Hotel, Motel, and Resort Desk Clerks
5310	Interviewers, Except Eligibility and Loan
5320	Library Assistants, Clerical
5330	Loan Interviewers and Clerks
5340	New Account Clerks
5350	Correspondent clerks and order clerks
5360	Human Resources Assistants, Except Payroll and Timekeeping
5400	Receptionists and Information Clerks
5410	Reservation and Transportation Ticket Agents and Travel Clerks
5420	Information and Record Clerks, All Other
5500	Cargo and Freight Agents
5510	Couriers and Messengers
5520	Dispatchers
5530	Meter Readers, Utilities

5540	Postal Service Clerks
5550	Postal Service Mail Carriers
5560	Postal Service Mail Sorters, Processors, and Processing Machine Operators
5600	Production, Planning, and Expediting Clerks
5610	Shipping, Receiving, and Traffic Clerks
5620	Stock Clerks and Order Fillers
5630	Weighers, Measurers, Checkers, and Samplers, Recordkeeping
5700	Secretaries and Administrative Assistants
5800	Computer Operators
5810	Data Entry Keyers
5820	Word Processors and Typists
5840	Insurance Claims and Policy Processing Clerks
5850	Mail Clerks and Mail Machine Operators, Except Postal Service
5860	Office Clerks, General
5900	Office Machine Operators, Except Computer
5910	Proofreaders and Copy Markers
5920	Statistical Assistants
5940	Office and administrative support workers, nec
6005	First-Line Supervisors of Farming, Fishing, and Forestry Workers
6010	Agricultural Inspectors
6040	Graders and Sorters, Agricultural Products
6050	Agricultural workers, nec
6100	Fishing and hunting workers
6120	Forest and Conservation Workers

6130	Logging Workers
6200	First-Line Supervisors of Construction Trades and Extraction Workers
6210	Boilermakers
6220	Brickmasons, Blockmasons, and Stonemasons
6230	Carpenters
6240	Carpet, Floor, and Tile Installers and Finishers
6250	Cement Masons, Concrete Finishers, and Terrazzo Workers
6260	Construction Laborers
6300	Paving, Surfacing, and Tamping Equipment Operators
6320	Construction equipment operators except paving, surfacing, and tamping equipment operators
6330	Drywall Installers, Ceiling Tile Installers, and Tapers
6355	Electricians
6360	Glaziers
6400	Insulation Workers
6420	Painters, Construction and Maintenance
6430	Paperhangers
6440	Pipelayers, Plumbers, Pipefitters, and Steamfitters
6460	Plasterers and Stucco Masons
6500	Reinforcing Iron and Rebar Workers
6515	Roofers
6520	Sheet Metal Workers, metal-working
6530	Structural Iron and Steel Workers
6600	Helpers, Construction Trades

6660	Construction and Building Inspectors
6700	Elevator Installers and Repairers
6710	Fence Erectors
6720	Hazardous Materials Removal Workers
6730	Highway Maintenance Workers
6740	Rail-Track Laying and Maintenance Equipment Operators
6765	Construction workers, nec
6800	Derrick, rotary drill, and service unit operators, and roustabouts, oil, gas, and mining
6820	Earth Drillers, Except Oil and Gas
6830	Explosives Workers, Ordnance Handling Experts, and Blasters
6840	Mining Machine Operators
6940	Extraction workers, nec
7000	First-Line Supervisors of Mechanics, Installers, and Repairers
7010	Computer, Automated Teller, and Office Machine Repairers
7020	Radio and Telecommunications Equipment Installers and Repairers
7030	Avionics Technicians
7040	Electric Motor, Power Tool, and Related Repairers
7100	Electrical and electronics repairers, transportation equipment, and industrial and utility
7110	Electronic Equipment Installers and Repairers, Motor Vehicles
7120	Electronic Home Entertainment Equipment Installers and Repairers
7125	Electronic Repairs, nec
7130	Security and Fire Alarm Systems Installers
7140	Aircraft Mechanics and Service Technicians
7150	Automotive Body and Related Repairers

7160	Automotive Glass Installers and Repairers
7200	Automotive Service Technicians and Mechanics
7210	Bus and Truck Mechanics and Diesel Engine Specialists
7220	Heavy Vehicle and Mobile Equipment Service Technicians and Mechanics
7240	Small Engine Mechanics
7260	Vehicle and Mobile Equipment Mechanics, Installers, and Repairers, nec
7300	Control and Valve Installers and Repairers
7315	Heating, Air Conditioning, and Refrigeration Mechanics and Installers
7320	Home Appliance Repairers
7330	Industrial and Refractory Machinery Mechanics
7340	Maintenance and Repair Workers, General
7350	Maintenance Workers, Machinery
7360	Millwrights
7410	Electrical Power-Line Installers and Repairers
7420	Telecommunications Line Installers and Repairers
7430	Precision Instrument and Equipment Repairers
7510	Coin, Vending, and Amusement Machine Servicers and Repairers
7540	Locksmiths and Safe Repairers
7550	Manufactured Building and Mobile Home Installers
7560	Riggers
7610	Helpers--Installation, Maintenance, and Repair Workers
7630	Other Installation, Maintenance, and Repair Workers Including Wind Turbine Service Technicians, and Commercial Divers, and Signal and Track Switch Repairers
7700	First-Line Supervisors of Production and Operating Workers

7710	Aircraft Structure, Surfaces, Rigging, and Systems Assemblers
7720	Electrical, Electronics, and Electromechanical Assemblers
7730	Engine and Other Machine Assemblers
7740	Structural Metal Fabricators and Fitters
7750	Assemblers and Fabricators, nec
7800	Bakers
7810	Butchers and Other Meat, Poultry, and Fish Processing Workers
7830	Food and Tobacco Roasting, Baking, and Drying Machine Operators and Tenders
7840	Food Batchmakers
7850	Food Cooking Machine Operators and Tenders
7855	Food Processing, nec
7900	Computer Control Programmers and Operators
7920	Extruding and Drawing Machine Setters, Operators, and Tenders, Metal and Plastic
7930	Forging Machine Setters, Operators, and Tenders, Metal and Plastic
7940	Rolling Machine Setters, Operators, and Tenders, metal and Plastic
7950	Cutting, Punching, and Press Machine Setters, Operators, and Tenders, Metal and Plastic
7960	Drilling and Boring Machine Tool Setters, Operators, and Tenders, Metal and Plastic
8000	Grinding, Lapping, Polishing, and Buffing Machine Tool Setters, Operators, and Tenders, Metal and Plastic
8010	Lathe and Turning Machine Tool Setters, Operators, and Tenders, Metal and Plastic
8030	Machinists
8040	Metal Furnace Operators, Tenders, Pourers, and Casters
8060	Model Makers and Patternmakers, Metal and Plastic
8100	Molders and Molding Machine Setters, Operators, and Tenders, Metal and Plastic

8130	Tool and Die Makers
8140	Welding, Soldering, and Brazing Workers
8150	Heat Treating Equipment Setters, Operators, and Tenders, Metal and Plastic
8200	Plating and Coating Machine Setters, Operators, and Tenders, Metal and Plastic
8210	Tool Grinders, Filers, and Sharpeners
8220	Metal workers and plastic workers, nec
8230	Bookbinders, Printing Machine Operators, and Job Printers
8250	Prepress Technicians and Workers
8300	Laundry and Dry-Cleaning Workers
8310	Pressers, Textile, Garment, and Related Materials
8320	Sewing Machine Operators
8330	Shoe and Leather Workers and Repairers
8340	Shoe Machine Operators and Tenders
8350	Tailors, Dressmakers, and Sewers
8400	Textile bleaching and dyeing, and cutting machine setters, operators, and tenders
8410	Textile Knitting and Weaving Machine Setters, Operators, and Tenders
8420	Textile Winding, Twisting, and Drawing Out Machine Setters, Operators, and Tenders
8450	Upholsterers
8460	Textile, Apparel, and Furnishings workers, nec
8500	Cabinetmakers and Bench Carpenters
8510	Furniture Finishers
8530	Sawing Machine Setters, Operators, and Tenders, Wood
8540	Woodworking Machine Setters, Operators, and Tenders, Except Sawing
8550	Woodworkers including model makers and patternmakers, nec

8600	Power Plant Operators, Distributors, and Dispatchers
8610	Stationary Engineers and Boiler Operators
8620	Water Wastewater Treatment Plant and System Operators
8630	Plant and System Operators, nec
8640	Chemical Processing Machine Setters, Operators, and Tenders
8650	Crushing, Grinding, Polishing, Mixing, and Blending Workers
8710	Cutting Workers
8720	Extruding, Forming, Pressing, and Compacting Machine Setters, Operators, and Tenders
8730	Furnace, Kiln, Oven, Drier, and Kettle Operators and Tenders
8740	Inspectors, Testers, Sorters, Samplers, and Weighers
8750	Jewelers and Precious Stone and Metal Workers
8760	Medical, Dental, and Ophthalmic Laboratory Technicians
8800	Packaging and Filling Machine Operators and Tenders
8810	Painting Workers and Dyers
8830	Photographic Process Workers and Processing Machine Operators
8850	Adhesive Bonding Machine Operators and Tenders
8860	Cleaning, Washing, and Metal Pickling Equipment Operators and Tenders
8910	Etchers, Engravers, and Lithographers
8920	Molders, Shapers, and Casters, Except Metal and Plastic
8930	Paper Goods Machine Setters, Operators, and Tenders
8940	Tire Builders
8950	Helpers--Production Workers
8965	Other production workers including semiconductor processors and cooling and freezing equipment operators

9000	Supervisors of Transportation and Material Moving Workers
9030	Aircraft Pilots and Flight Engineers
9040	Air Traffic Controllers and Airfield Operations Specialists
9050	Flight Attendants and Transportation Workers and Attendants
9100	Bus and Ambulance Drivers and Attendants
9130	Driver/Sales Workers and Truck Drivers
9140	Taxi Drivers and Chauffeurs
9150	Motor Vehicle Operators, All Other
9200	Locomotive Engineers and Operators
9230	Railroad Brake, Signal, and Switch Operators
9240	Railroad Conductors and Yardmasters
9260	Subway, Streetcar, and Other Rail Transportation Workers
9300	Sailors and marine oilers, and ship engineers
9310	Ship and Boat Captains and Operators
9350	Parking Lot Attendants
9360	Automotive and Watercraft Service Attendants
9410	Transportation Inspectors
9420	Transportation workers, nec
9510	Crane and Tower Operators
9520	Dredge, Excavating, and Loading Machine Operators
9560	Conveyor operators and tenders, and hoist and winch operators
9600	Industrial Truck and Tractor Operators
9610	Cleaners of Vehicles and Equipment
9620	Laborers and Freight, Stock, and Material Movers, Hand

9630	Machine Feeders and Offbearers
9640	Packers and Packagers, Hand
9650	Pumping Station Operators
9720	Refuse and Recyclable Material Collectors
9750	Material moving workers, nec
9800	Military Officer Special and Tactical Operations Leaders
9810	First-Line Enlisted Military Supervisors
9820	Military Enlisted Tactical Operations and Air/Weapons Specialists and Crew Members
9830	Military, Rank Not Specified
9920	Unemployed, with No Work Experience in the Last 5 Years or Earlier or Never Worked
9999	Unknown

Variable: "OCC1990"

Name:	OCC1990
Label:	Occupation, 1990 basis
Variable Text:	OCC1990 is a modified version of the 1990 Census Bureau occupational classification scheme. Like OCC1950, OCC1990 offers researchers a consistent long-term classification of occupations.<br><br>The occupational coding scheme in CPS data has changed several times since the 1960s. The CPS's occupational coding scheme has mirrored that of the Census Bureau, though the CPS has always introduced major coding changes one-to-three years later than the Census Bureau. All original occupational information is stored in the OCC variable. The meaning of codes in the OCC variable changes with each new coding scheme.<br><br>We chose the 1990 scheme as the standard for OCC1990 so that no year's occupational data would be forced to bridge both of the two most significant changes in census-based coding schemes: from 1970 to 1980 and from 1990 to 2000. In OCC1990, all samples from 1968 onwards bridge no more than one of these major shifts. For this reason, the variable may be preferable to OCC1950 for the samples from 1980 onward. Sensitivity testing suggests that OCC1990 performs very similarly to OCC1950 for most purposes.<br><br>The original 1990 occupational scheme [URL omitted from DDI.] has 514 categories. OCC1990 combines a number of occupational categories to maximize the variable's consistency over time. The resulting OCC1990 classification scheme contains 389 categories (see the "Codes and Frequencies" link above). Many users will want to further aggregate categories into the broad occupational categories implicit in the 1990 scheme: Managerial and Professional (000-200); Technical, Sales, and Administrative (201-400);

	Service (401-470); Farming, Forestry, and Fishing (471-500); Precision Production, Craft, and Repairers (501-700); Operatives and Laborers (701-900); Non-occupational responses (900-999).<br><br>OCC1990 was created using a series of technical papers published by the Census Bureau shortly after each census was administered. These papers provide detailed analyses of how the occupational coding scheme for each census year differed from the scheme used during the previous census year. These occupational "crosswalks" are based on samples of cases that are "double coded" into the occupational schemes of the current and previous census year. The original Census Bureau crosswalks are available via links in "Occupation and Industry Variables" [URL omitted from DDI.] of the IPUMS-USA documentation.<br><br>Using the information from the occupational crosswalks, we traced the proportion of each occupation as it broke out into more specific occupations or as it was combined with others into a more general occupation. To take one example from the technical paper produced after the 2000 census: of persons coded as "Gaming managers" in 2000 (2000 code 33), the Census Bureau determined that 35% would have been coded as "Managers, service organizations" in 1990 (1990 code 21), while 65% would have been coded as "Managers, food serving and lodging establishments" (1990 code 17). Thus, OCC1990 assigns a code of 17 to the cases in the 2000 IPUMS sample having an original 2000 OCC value of 33. We generated the same information for every occupational code in every census year from 1960-2000.<br><br>Researchers at the Bureau of Labor Statistics (BLS) then used the resulting tables to create aggregated occupational categories that were more useful for long-term analyses. We have performed a variety of tests to ensure that the new categories are as robust as possible over the long-term. More specifics on their methods and a detailed comparison of OCC1950 and OCC1990 can be found in the BLS Working Paper [URL omitted from DDI.] on the topic.
Concept:	Work Variables -- PERSON
Start Position:	131
End Position:	133
Width:	3
Variable Format:	numeric
Implied Decimal Places:	0

Categories

Value	Label
003	Legislators

004	Chief executives and public administrators
007	Financial managers
008	Human resources and labor relations managers
013	Managers and specialists in marketing, advertising, and public relations
014	Managers in education and related fields
015	Managers of medicine and health occupations
016	Postmasters and mail superintendents
017	Managers of food-serving and lodging establishments
018	Managers of properties and real estate
019	Funeral directors
021	Managers of service organizations, n.e.c.
022	Managers and administrators, n.e.c.
023	Accountants and auditors
024	Insurance underwriters
025	Other financial specialists
026	Management analysts
027	Personnel, HR, training, and labor relations specialists
028	Purchasing agents and buyers, of farm products
029	Buyers, wholesale and retail trade
033	Purchasing managers, agents and buyers, n.e.c.
034	Business and promotion agents
035	Construction inspectors
036	Inspectors and compliance officers, outside construction
037	Management support occupations

043	Architects
044	Aerospace engineer
045	Metallurgical and materials engineers, variously phrased
047	Petroleum, mining, and geological engineers
048	Chemical engineers
053	Civil engineers
055	Electrical engineer
056	Industrial engineers
057	Mechanical engineers
059	Not-elsewhere-classified engineers
064	Computer systems analysts and computer scientists
065	Operations and systems researchers and analysts
066	Actuaries
067	Statisticians
068	Mathematicians and mathematical scientists
069	Physicists and astronomers
073	Chemists
074	Atmospheric and space scientists
075	Geologists
076	Physical scientists, n.e.c.
077	Agricultural and food scientists
078	Biological scientists
079	Foresters and conservation scientists
083	Medical scientists

084	Physicians
085	Dentists
086	Veterinarians
087	Optometrists
088	Podiatrists
089	Other health and therapy
095	Registered nurses
096	Pharmacists
097	Dietitians and nutritionists
098	Respiratory therapists
099	Occupational therapists
103	Physical therapists
104	Speech therapists
105	Therapists, n.e.c.
106	Physicians' assistants
113	Earth, environmental, and marine science instructors
114	Biological science instructors
115	Chemistry instructors
116	Physics instructors
118	Psychology instructors
119	Economics instructors
123	History instructors
125	Sociology instructors
127	Engineering instructors

128	Math instructors
139	Education instructors
145	Law instructors
147	Theology instructors
149	Home economics instructors
150	Humanities profs/instructors, college, nec
154	Subject instructors (HS/college)
155	Kindergarten and earlier school teachers
156	Primary school teachers
157	Secondary school teachers
158	Special education teachers
159	Teachers , n.e.c.
163	Vocational and educational counselors
164	Librarians
165	Archivists and curators
166	Economists, market researchers, and survey researchers
167	Psychologists
168	Sociologists
169	Social scientists, n.e.c.
173	Urban and regional planners
174	Social workers
175	Recreation workers
176	Clergy and religious workers
178	Lawyers

179	Judges
183	Writers and authors
184	Technical writers
185	Designers
186	Musician or composer
187	Actors, directors, producers
188	Art makers: painters, sculptors, craft-artists, and print-makers
189	Photographers
193	Dancers
194	Art/entertainment performers and related
195	Editors and reporters
198	Announcers
199	Athletes, sports instructors, and officials
200	Professionals, n.e.c.
203	Clinical laboratory technologies and technicians
204	Dental hygenists
205	Health record tech specialists
206	Radiologic tech specialists
207	Licensed practical nurses
208	Health technologists and technicians, n.e.c.
213	Electrical and electronic (engineering) technicians
214	Engineering technicians, n.e.c.
215	Mechanical engineering technicians
217	Drafters

218	Surveyors, cartographers, mapping scientists and technicians
223	Biological technicians
224	Chemical technicians
225	Other science technicians
226	Airplane pilots and navigators
227	Air traffic controllers
228	Broadcast equipment operators
229	Computer software developers
233	Programmers of numerically controlled machine tools
234	Legal assistants, paralegals, legal support, etc
235	Technicians, n.e.c.
243	Supervisors and proprietors of sales jobs
253	Insurance sales occupations
254	Real estate sales occupations
255	Financial services sales occupations
256	Advertising and related sales jobs
258	Sales engineers
274	Salespersons, n.e.c.
275	Retail sales clerks
276	Cashiers
277	Door-to-door sales, street sales, and news vendors
283	Sales demonstrators / promoters / models
290	Sales workers--allocated (1990 internal census)
303	Office supervisors

308	Computer and peripheral equipment operators
313	Secretaries
314	Stenographers
315	Typists
316	Interviewers, enumerators, and surveyors
317	Hotel clerks
318	Transportation ticket and reservation agents
319	Receptionists
323	Information clerks, nec
326	Correspondence and order clerks
328	Human resources clerks, except payroll and timekeeping
329	Library assistants
335	File clerks
336	Records clerks
337	Bookkeepers and accounting and auditing clerks
338	Payroll and timekeeping clerks
343	Cost and rate clerks (financial records processing)
344	Billing clerks and related financial records processing
345	Duplication machine operators / office machine operators
346	Mail and paper handlers
347	Office machine operators, n.e.c.
348	Telephone operators
349	Other telecom operators
354	Postal clerks, excluding mail carriers

355	Mail carriers for postal service
356	Mail clerks, outside of post office
357	Messengers
359	Dispatchers
361	Inspectors, n.e.c.
364	Shipping and receiving clerks
365	Stock and inventory clerks
366	Meter readers
368	Weighers, measurers, and checkers
373	Material recording, scheduling, production, planning, and expediting clerks
375	Insurance adjusters, examiners, and investigators
376	Customer service reps, investigators and adjusters, except insurance
377	Eligibility clerks for government programs; social welfare
378	Bill and account collectors
379	General office clerks
383	Bank tellers
384	Proofreaders
385	Data entry keyers
386	Statistical clerks
387	Teacher's aides
389	Administrative support jobs, n.e.c.
390	Professional, technical, and kindred workers--allocated (1990 internal census)
391	Clerical and kindred workers--allocated (1990 internal census)
405	Housekeepers, maids, butlers, stewards, and lodging quarters cleaners

407	Private household cleaners and servants
408	Private household workers--allocated (1990 internal census)
415	Supervisors of guards
417	Fire fighting, prevention, and inspection
418	Police, detectives, and private investigators
423	Other law enforcement: sheriffs, bailiffs, correctional institution officers
425	Crossing guards and bridge tenders
426	Guards, watchmen, doorkeepers
427	Protective services, n.e.c.
434	Bartenders
435	Waiter/waitress
436	Cooks, variously defined
438	Food counter and fountain workers
439	Kitchen workers
443	Waiter's assistant
444	Misc food prep workers
445	Dental assistants
446	Health aides, except nursing
447	Nursing aides, orderlies, and attendants
448	Supervisors of cleaning and building service
453	Janitors
454	Elevator operators
455	Pest control occupations
456	Supervisors of personal service jobs, n.e.c.

457	Barbers
458	Hairdressers and cosmetologists
459	Recreation facility attendants
461	Guides
462	Ushers
463	Public transportation attendants and inspectors
464	Baggage porters
465	Welfare service aides
468	Child care workers
469	Personal service occupations, nec
473	Farmers (owners and tenants)
474	Horticultural specialty farmers
475	Farm managers, except for horticultural farms
476	Managers of horticultural specialty farms
479	Farm workers
480	Farm laborers and farm foreman--allocated (1990 internal census)
483	Marine life cultivation workers
484	Nursery farming workers
485	Supervisors of agricultural occupations
486	Gardeners and groundskeepers
487	Animal caretakers except on farms
488	Graders and sorters of agricultural products
489	Inspectors of agricultural products
496	Timber, logging, and forestry workers

498	Fishers, hunters, and kindred
503	Supervisors of mechanics and repairers
505	Automobile mechanics
507	Bus, truck, and stationary engine mechanics
508	Aircraft mechanics
509	Small engine repairers
514	Auto body repairers
516	Heavy equipment and farm equipment mechanics
518	Industrial machinery repairers
519	Machinery maintenance occupations
523	Repairers of industrial electrical equipment
525	Repairers of data processing equipment
526	Repairers of household appliances and power tools
527	Telecom and line installers and repairers
533	Repairers of electrical equipment, n.e.c.
534	Heating, air conditioning, and refigeration mechanics
535	Precision makers, repairers, and smiths
536	Locksmiths and safe repairers
538	Office machine repairers and mechanics
539	Repairers of mechanical controls and valves
543	Elevator installers and repairers
544	Millwrights
549	Mechanics and repairers, n.e.c.
558	Supervisors of construction work

563	Masons, tilers, and carpet installers
567	Carpenters
573	Drywall installers
575	Electricians
577	Electric power installers and repairers
579	Painters, construction and maintenance
583	Paperhangers
584	Plasterers
585	Plumbers, pipe fitters, and steamfitters
588	Concrete and cement workers
589	Glaziers
593	Insulation workers
594	Paving, surfacing, and tamping equipment operators
595	Roofers and slaters
596	Sheet metal duct installers
597	Structural metal workers
598	Drillers of earth
599	Construction trades, n.e.c.
614	Drillers of oil wells
615	Explosives workers
616	Miners
617	Other mining occupations
628	Production supervisors or foremen
634	Tool and die makers and die setters

637	Machinists
643	Boilermakers
644	Precision grinders and filers
645	Patternmakers and model makers
646	Lay-out workers
649	Engravers
653	Tinsmiths, coppersmiths, and sheet metal workers
657	Cabinetmakers and bench carpenters
658	Furniture and wood finishers
659	Other precision woodworkers
666	Dressmakers and seamstresses
667	Tailors
668	Upholsterers
669	Shoe repairers
674	Other precision apparel and fabric workers
675	Hand molders and shapers, except jewelers
677	Optical goods workers
678	Dental laboratory and medical appliance technicians
679	Bookbinders
684	Other precision and craft workers
686	Butchers and meat cutters
687	Bakers
688	Batch food makers
693	Adjusters and calibrators

694	Water and sewage treatment plant operators
695	Power plant operators
696	Plant and system operators, stationary engineers
699	Other plant and system operators
703	Lathe, milling, and turning machine operatives
706	Punching and stamping press operatives
707	Rollers, roll hands, and finishers of metal
708	Drilling and boring machine operators
709	Grinding, abrading, buffing, and polishing workers
713	Forge and hammer operators
717	Fabricating machine operators, n.e.c.
719	Molders, and casting machine operators
723	Metal platers
724	Heat treating equipment operators
726	Wood lathe, routing, and planing machine operators
727	Sawing machine operators and sawyers
728	Shaping and joining machine operator (woodworking)
729	Nail and tacking machine operators (woodworking)
733	Other woodworking machine operators
734	Printing machine operators, n.e.c.
735	Photoengravers and lithographers
736	Typesetters and compositors
738	Winding and twisting textile/apparel operatives
739	Knitters, loopers, and toppers textile operatives

743	Textile cutting machine operators
744	Textile sewing machine operators
745	Shoemaking machine operators
747	Pressing machine operators (clothing)
748	Laundry workers
749	Misc textile machine operators
753	Cementing and gluing maching operators
754	Packers, fillers, and wrappers
755	Extruding and forming machine operators
756	Mixing and blending machine operatives
757	Separating, filtering, and clarifying machine operators
759	Painting machine operators
763	Roasting and baking machine operators (food)
764	Washing, cleaning, and pickling machine operators
765	Paper folding machine operators
766	Furnace, kiln, and oven operators, apart from food
768	Crushing and grinding machine operators
769	Slicing and cutting machine operators
773	Motion picture projectionists
774	Photographic process workers
779	Machine operators, n.e.c.
783	Welders and metal cutters
784	Solderers
785	Assemblers of electrical equipment

789	Hand painting, coating, and decorating occupations
796	Production checkers and inspectors
799	Graders and sorters in manufacturing
803	Supervisors of motor vehicle transportation
804	Truck, delivery, and tractor drivers
808	Bus drivers
809	Taxi cab drivers and chauffeurs
813	Parking lot attendants
815	Transport equipment operatives--allocated (1990 internal census)
823	Railroad conductors and yardmasters
824	Locomotive operators (engineers and firemen)
825	Railroad brake, coupler, and switch operators
829	Ship crews and marine engineers
834	Water transport infrastructure tenders and crossing guards
844	Operating engineers of construction equipment
848	Crane, derrick, winch, and hoist operators
853	Excavating and loading machine operators
859	Misc material moving occupations
865	Helpers, constructions
866	Helpers, surveyors
869	Construction laborers
874	Production helpers
875	Garbage and recyclable material collectors
876	Materials movers: stevedores and longshore workers

877	Stock handlers
878	Machine feeders and offbearers
883	Freight, stock, and materials handlers
885	Garage and service station related occupations
887	Vehicle washers and equipment cleaners
888	Packers and packagers by hand
889	Laborers outside construction
890	Laborers, except farm--allocated (1990 internal census)
905	Military
991	Unemployed
999	Unknown

Variable: "IND1990"

Name:	IND1990
Label:	Industry, 1990 basis
Variable Text:	IND1990 recodes information contained in the variable IND into the 1990 Census Bureau industrial classification system. Developed to enhance the comparability of industry data in historical U.S. census samples in IPUMS-USA, IND1990 also provides a consistent set of industry codes for IPUMS-CPS from 1968 forward.<br><br>For general discussion of the IND1990 variable, users should consult the IPUMS-USA documentation. For discussion of the CPS data on industry that are recoded into IND1990, see IND.
Concept:	Work Variables -- PERSON
Start Position:	134
End Position:	136
Width:	3

Variable Format:	numeric
Implied Decimal Places:	0

Categories

Value	Label
000	NIU
010	Agricultural production, crops
011	Agricultural production, livestock
012	Veterinary services
020	Landscape and horticultural services
030	Agricultural services, n.e.c.
031	Forestry
032	Fishing, hunting, and trapping
040	Metal mining
041	Coal mining
042	Oil and gas extraction
050	Nonmetallic mining and quarrying, except fuels
060	All construction
100	Meat products
101	Dairy products
102	Canned, frozen, and preserved fruits and vegetables
110	Grain mill products
111	Bakery products

112	Sugar and confectionery products
120	Beverage industries
121	Misc. food preparations and kindred products
122	Food industries, n.s.
130	Tobacco manufactures
132	Knitting mills
140	Dyeing and finishing textiles, except wool and knit goods
141	Carpets and rugs
142	Yarn, thread, and fabric mills
150	Miscellaneous textile mill products
151	Apparel and accessories, except knit
152	Miscellaneous fabricated textile products
160	Pulp, paper, and paperboard mills
161	Miscellaneous paper and pulp products
162	Paperboard containers and boxes
171	Newspaper publishing and printing
172	Printing, publishing, and allied industries, except newspapers
180	Plastics, synthetics, and resins
181	Drugs
182	Soaps and cosmetics
190	Paints, varnishes, and related products
191	Agricultural chemicals
192	Industrial and miscellaneous chemicals
200	Petroleum refining

201	Miscellaneous petroleum and coal products
210	Tires and inner tubes
211	Other rubber products, and plastics footwear and belting
212	Miscellaneous plastics products
220	Leather tanning and finishing
221	Footwear, except rubber and plastic
222	Leather products, except footwear
229	Manufacturing, non-durable - allocated
230	Logging
231	Sawmills, planing mills, and millwork
232	Wood buildings and mobile homes
241	Miscellaneous wood products
242	Furniture and fixtures
250	Glass and glass products
251	Cement, concrete, gypsum, and plaster products
252	Structural clay products
261	Pottery and related products
262	Misc. nonmetallic mineral and stone products
270	Blast furnaces, steelworks, rolling and finishing mills
271	Iron and steel foundries
272	Primary aluminum industries
280	Other primary metal industries
281	Cutlery, handtools, and general hardware
282	Fabricated structural metal products

290	Screw machine products
291	Metal forgings and stampings
292	Ordnance
300	Miscellaneous fabricated metal products
301	Metal industries, n.s.
310	Engines and turbines
311	Farm machinery and equipment
312	Construction and material handling machines
320	Metalworking machinery
321	Office and accounting machines
322	Computers and related equipment
331	Machinery, except electrical, n.e.c.
332	Machinery, n.s.
340	Household appliances
341	Radio, TV, and communication equipment
342	Electrical machinery, equipment, and supplies, n.e.c.
350	Electrical machinery, equipment, and supplies, n.s.
351	Motor vehicles and motor vehicle equipment
352	Aircraft and parts
360	Ship and boat building and repairing
361	Railroad locomotives and equipment
362	Guided missiles, space vehicles, and parts
370	Cycles and miscellaneous transportation equipment
371	Scientific and controlling instruments

372	Medical, dental, and optical instruments and supplies
380	Photographic equipment and supplies
381	Watches, clocks, and clockwork operated devices
390	Toys, amusement, and sporting goods
391	Miscellaneous manufacturing industries
392	Manufacturing industries, n.s.
400	Railroads
401	Bus service and urban transit
402	Taxicab service
410	Trucking service
411	Warehousing and storage
412	U.S. Postal Service
420	Water transportation
421	Air transportation
422	Pipe lines, except natural gas
432	Services incidental to transportation
440	Radio and television broadcasting and cable
441	Wired communications
442	Telegraph and miscellaneous communications services
450	Electric light and power
451	Gas and steam supply systems
452	Electric and gas, and other combinations
470	Water supply and irrigation
471	Sanitary services

472	Utilities, n.s.
500	Motor vehicles and equipment
501	Furniture and home furnishings
502	Lumber and construction materials
510	Professional and commercial equipment and supplies
511	Metals and minerals, except petroleum
512	Electrical goods
521	Hardware, plumbing and heating supplies
530	Machinery, equipment, and supplies
531	Scrap and waste materials
532	Miscellaneous wholesale, durable goods
540	Paper and paper products
541	Drugs, chemicals, and allied products
542	Apparel, fabrics, and notions
550	Groceries and related products
551	Farm-product raw materials
552	Petroleum products
560	Alcoholic beverages
561	Farm supplies
562	Miscellaneous wholesale, nondurable goods
571	Wholesale trade, n.s.
580	Lumber and building material retailing
581	Hardware stores
582	Retail nurseries and garden stores

590	Mobile home dealers
591	Department stores
592	Variety stores
600	Miscellaneous general merchandise stores
601	Grocery stores
602	Dairy products stores
610	Retail bakeries
611	Food stores, n.e.c.
612	Motor vehicle dealers
620	Auto and home supply stores
621	Gasoline service stations
622	Miscellaneous vehicle dealers
623	Apparel and accessory stores, except shoe
630	Shoe stores
631	Furniture and home furnishings stores
632	Household appliance stores
633	Radio, TV, and computer stores
640	Music stores
641	Eating and drinking places
642	Drug stores
650	Liquor stores
651	Sporting goods, bicycles, and hobby stores
652	Book and stationery stores
660	Jewelry stores

661	Gift, novelty, and souvenir shops
662	Sewing, needlework, and piece goods stores
663	Catalog and mail order houses
670	Vending machine operators
671	Direct selling establishments
672	Fuel dealers
681	Retail florists
682	Miscellaneous retail stores
691	Retail trade, n.s.
700	Banking
701	Savings institutions, including credit unions
702	Credit agencies, n.e.c.
710	Security, commodity brokerage, and investment companies
711	Insurance
712	Real estate, including real estate-insurance offices
721	Advertising
722	Services to dwellings and other buildings
731	Personnel supply services
732	Computer and data processing services
740	Detective and protective services
741	Business services, n.e.c.
742	Automotive rental and leasing, without drivers
750	Automobile parking and carwashes
751	Automotive repair and related services

752	Electrical repair shops
760	Miscellaneous repair services
761	Private households
762	Hotels and motels
770	Lodging places, except hotels and motels
771	Laundry, cleaning, and garment services
772	Beauty shops
780	Barber shops
781	Funeral service and crematories
782	Shoe repair shops
790	Dressmaking shops
791	Miscellaneous personal services
800	Theaters and motion pictures
801	Video tape rental
802	Bowling centers
810	Miscellaneous entertainment and recreation services
812	Offices and clinics of physicians
820	Offices and clinics of dentists
821	Offices and clinics of chiropractors
822	Offices and clinics of optometrists
830	Offices and clinics of health practitioners, n.e.c.
831	Hospitals
832	Nursing and personal care facilities
840	Health services, n.e.c.

841	Legal services
842	Elementary and secondary schools
850	Colleges and universities
851	Vocational schools
852	Libraries
860	Educational services, n.e.c.
861	Job training and vocational rehabilitation services
862	Child day care services
863	Family child care homes
870	Residential care facilities, without nursing
871	Social services, n.e.c.
872	Museums, art galleries, and zoos
873	Labor unions
880	Religious organizations
881	Membership organizations, n.e.c.
882	Engineering, architectural, and surveying services
890	Accounting, auditing, and bookkeeping services
891	Research, development, and testing services
892	Management and public relations services
893	Miscellaneous professional and related services
900	Executive and legislative offices
901	General government, n.e.c.
910	Justice, public order, and safety
921	Public finance, taxation, and monetary policy

922	Administration of human resources programs
930	Administration of environmental quality and housing programs
931	Administration of economic programs
932	National security and international affairs
940	Army
941	Air Force
942	Navy
950	Marines
951	Coast Guard
952	Armed Forces, branch not specified
960	Military Reserves or National Guard
998	Unknown

Variable: "OCC1950"

Name:	OCC1950
Label:	Occupation, 1950 basis
Variable Text:	OCC1950 recodes information contained in the variable OCC into the 1950 Census Bureau occupational classification system. Developed to enhance the comparability of occupational data in historical U.S. census samples in IPUMS-USA, OCC1950 also provides a consistent set of occupational codes for IPUMS-CPS from 1968 forward.<br><br>For general discussion of the OCC1950 variable, users should consult the IPUMS-USA documentation. For discussion of the CPS data on occupations that are recoded into OCC1950, see the OCC variable.
Concept:	Work Variables -- PERSON
Start Position:	137
End Position:	139
Width:	3

Variable Format:	numeric
Implied Decimal Places:	0

Categories

Value	Label
000	Accountants and auditors
001	Actors and actresses
002	Airplane pilots and navigators
003	Architects
004	Artists and art teachers
005	Athletes
006	Authors
007	Chemists
008	Chiropractors
009	Clergymen
010	College presidents and deans
012	Agricultural sciences
013	Biological sciences
014	Chemistry
015	Economics
016	Engineering
017	Geology and geophysics
018	Mathematics

019	Medical sciences
023	Physics
024	Psychology
025	Statistics
026	Natural science (n.e.c.)
027	Social sciences (n.e.c.)
028	Nonscientific subjects
029	Subject not specified
031	Dancers and dancing teachers
032	Dentists
033	Designers
034	Dieticians and nutritionists
035	Draftsmen
036	Editors and reporters
041	Engineers, aeronautical
042	Engineers, chemical
043	Engineers, civil
044	Engineers, electrical
045	Engineers, industrial
046	Engineers, mechanical
047	Engineers, metallurgical, metallurgists
048	Engineers, mining
049	Engineers (n.e.c.)
051	Entertainers (n.e.c.)

052	Farm and home management advisors
053	Foresters and conservationists
054	Funeral directors and embalmers
055	Lawyers and judges
056	Librarians
057	Musicians and music teachers
058	Nurses, professional
059	Nurses, student professional
061	Agricultural scientists
062	Biological scientists
063	Geologists and geophysicists
067	Mathematicians
068	Physicists
069	Miscellaneous natural scientists
070	Optometrists
071	Osteopaths
072	Personnel and labor relations workers
073	Pharmacists
074	Photographers
075	Physicians and surgeons
076	Radio operators
077	Recreation and group workers
078	Religious workers
079	Social and welfare workers, except group

081	Economists
082	Psychologists
083	Statisticians and actuaries
084	Miscellaneous social scientists
091	Sports instructors and officials
092	Surveyors
093	Teachers (n.e.c.)
094	Technicians, medical and dental
095	Technicians, testing
096	Technicians (n.e.c.)
097	Therapists and healers (n.e.c.)
098	Veterinarians
099	Professional, technical and kindred workers (n.e.c.)
100	Farmers (owners and tenants)
123	Farm managers
200	Buyers and department heads, store
201	Buyers and shippers, farm products
203	Conductors, railroad
204	Credit men
205	Floormen and floor managers, store
210	Inspectors, public administration
230	Managers and superintendents, building
240	Officers, pilots, pursers and engineers, ship
250	Officials and administrators (n.e.c.), public administration

260	Officials, lodge, society, union, etc.
270	Postmasters
280	Purchasing agents and buyers (n.e.c.)
290	Managers, officials, and proprietors (n.e.c.)
300	Agents (n.e.c.)
301	Attendants and assistants, library
302	Attendants, physicians and dentists office
304	Baggagemen, transportation
305	Bank tellers
310	Bookkeepers
320	Cashiers
321	Collectors, bill and account
322	Dispatchers and starters, vehicle
325	Express messengers and railway mail clerks
335	Mail carriers
340	Messengers and office boys
341	Office machine operators
342	Shipping and receiving clerks
350	Stenographers, typists, and secretaries
360	Telegraph messengers
365	Telegraph operators
370	Telephone operators
380	Ticket, station, and express agents
390	Clerical and kindred workers (n.e.c.)

400	Advertising agents and salesmen
410	Auctioneers
420	Demonstrators
430	Hucksters and peddlers
450	Insurance agents and brokers
460	Newsboys
470	Real estate agents and brokers
480	Stock and bond salesmen
490	Salesmen and sales clerks (n.e.c.)
500	Bakers
501	Blacksmiths
502	Bookbinders
503	Boilermakers
504	Brickmasons, stonemasons, and tile setters
505	Cabinetmakers
510	Carpenters
511	Cement and concrete finishers
512	Compositors and typesetters
513	Cranemen, derrickmen, and hoistmen
514	Decorators and window dressers
515	Electricians
520	Electrotypers and stereotypers
521	Engravers, except photoengravers
522	Excavating, grading, and road machinery operators

523	Foremen (n.e.c.)
524	Forgemen and hammermen
525	Furriers
530	Glaziers
531	Heat treaters, annealers, temperers
532	Inspectors, scalers, and graders, log and lumber
533	Inspectors (n.e.c.)
534	Jewelers, watchmakers, goldsmiths, and silversmiths
535	Job setters, metal
540	Linemen and servicemen, telegraph, telephone, and power
541	Locomotive engineers
542	Locomotive firemen
543	Loom fixers
544	Machinists
545	Mechanics and repairmen, airplane
550	Mechanics and repairmen, automobile
551	Mechanics and repairmen, office machine
552	Mechanics and repairmen, radio and television
553	Mechanics and repairmen, railroad and car shop
554	Mechanics and repairmen (n.e.c.)
555	Millers, grain, flour, feed, etc.
560	Millwrights
561	Molders, metal
562	Motion picture projectionists

563	Opticians and lens grinders and polishers
564	Painters, construction and maintenance
565	Paperhangers
570	Pattern and model makers, except paper
571	Photoengravers and lithographers
572	Piano and organ tuners and repairmen
573	Plasterers
574	Plumbers and pipe fitters
575	Pressmen and plate printers, printing
580	Rollers and roll hands, metal
581	Roofers and slaters
582	Shoemakers and repairers, except factory
583	Stationary engineers
584	Stone cutters and stone carvers
585	Structural metal workers
590	Tailors and tailoresses
591	Tinsmiths, coppersmiths, and sheet metal workers
592	Tool makers, and die makers and setters
593	Upholsterers
594	Craftsmen and kindred workers (n.e.c.)
595	Members of the armed services
600	Apprentice auto mechanics
601	Apprentice bricklayers and masons
602	Apprentice carpenters

603	Apprentice electricians
604	Apprentice machinists and toolmakers
605	Apprentice mechanics, except auto
610	Apprentice plumbers and pipe fitters
611	Apprentices, building trades (n.e.c.)
612	Apprentices, metalworking trades (n.e.c.)
613	Apprentices, printing trades
614	Apprentices, other specified trades
615	Apprentices, trade not specified
620	Asbestos and insulation workers
621	Attendants, auto service and parking
622	Blasters and powdermen
623	Boatmen, canalmen, and lock keepers
624	Brakemen, railroad
625	Bus drivers
630	Chainmen, rodmen, and axmen, surveying
631	Conductors, bus and street railway
632	Deliverymen and routemen
633	Dressmakers and seamstresses, except factory
634	Dyers
635	Filers, grinders, and polishers, metal
640	Fruit, nut, veg graders and packers, except factory
641	Furnacemen, smeltermen and pourers
642	Heaters, metal

643	Laundry and dry cleaning operatives
644	Meat cutters, except slaughter and packing house
645	Milliners
650	Mine operatives and laborers
660	Motormen, mine, factory, logging camp, etc.
661	Motormen, street, subway, and elevated railway
662	Oilers and greaser, except auto
670	Painters, except construction or maintenance
671	Photographic process workers
672	Power station operators
673	Sailors and deck hands
674	Sawyers
675	Spinners, textile
680	Stationary firemen
681	Switchmen, railroad
682	Taxicab drivers and chauffers
683	Truck and tractor drivers
684	Weavers, textile
685	Welders and flame cutters
690	Operative and kindred workers (n.e.c.)
700	Housekeepers, private household
710	Laundressses, private household
720	Private household workers (n.e.c.)
730	Attendants, hospital and other institution

731	Attendants, professional and personal service (n.e.c.)
732	Attendants, recreation and amusement
740	Barbers, beauticians, and manicurists
750	Bartenders
751	Bootblacks
752	Boarding and lodging house keepers
753	Charwomen and cleaners
754	Cooks, except private household
760	Counter and fountain workers
761	Elevator operators
762	Firemen, fire protection
763	Guards, watchmen, and doorkeepers
764	Housekeepers and stewards, except private household
770	Janitors and sextons
771	Marshals and constables
772	Midwives
773	Policemen and detectives
780	Porters
781	Practical nurses
782	Sheriffs and bailiffs
783	Ushers, recreation and amusement
784	Waiters and waitresses
785	Watchmen (crossing) and bridge tenders
790	Service workers, except private household (n.e.c.)

810	Farm foremen
820	Farm laborers, wage workers
830	Farm laborers, unpaid family workers
840	Farm service laborers, self-employed
910	Fishermen and oystermen
920	Garage laborers and car washers and greasers
930	Gardeners, except farm, and groundskeepers
940	Longshoremen and stevedores
950	Lumbermen, raftsmen, and woodchoppers
960	Teamsters
970	Laborers (n.e.c.)
997	Unknown
999	Unemployed- last worked over x years ago

Variable: "IND"

Name:	IND
Label:	Industry
Variable Text:	IND reports the type of industry in which the person performed his or her primary occupation, which is recorded in the variables OCC (Occupation) and, after 1968, OCC1950 (Occupation, 1950 basis). "Industry" refers to the work setting and economic sector, while "occupation" relates to the worker's specific technical function.<br><br>For persons who were employed at the time of the survey, IND relates to the industrial sector in which the respondent worked during the preceding week. For unemployed persons and those not currently in the labor force, IND characterizes the industrial sector of the respondent's most recent job. The CPS interviewer collected information by asking what kind of work the person was doing, and Census Bureau staff coded the information into the CPS or census industrial classification. Researchers who wish to work with a consistent industrial coding scheme for 1968 forward should use the IND1950 variable. For general discussion of employment concepts, including the definition of those not in the labor force, see the documentation on EMPSTAT.
Concept:	Work Variables -- PERSON

Start Position:	140
End Position:	143
Width:	4
Variable Format:	numeric
Implied Decimal Places:	0
Coder Instructions:	IND is a 4-digit numeric variable.<br>(Codes for 1962-1967 are 2 digits; each is preceded by two zeroes in the first positions.)<br>(Codes for 1968-2002 are 3 digits; each is preceded by a zero in the first position.)<br><br>1962 [URL omitted from DDI.]<br>1963-1967 [URL omitted from DDI.]<br>1968-1970 [URL omitted from DDI.]<br>1971-1982 [URL omitted from DDI.]<br>1983-1991 [URL omitted from DDI.]<br>1992-2002 [URL omitted from DDI.]<br>2003-2008 [URL omitted from DDI.]<br>2009-2013 [URL omitted from DDI.]<br>2014-onward [URL omitted from DDI.]

Variable: "IND1950"

Name:	IND1950
Label:	Industry, 1950 basis
Variable Text:	IND1950 recodes information contained in the variable IND into the 1950 Census Bureau industrial classification system. Developed to enhance the comparability of industry data in historical U.S. census samples in IPUMS-USA, IND1950 also provides a consistent set of industry codes for IPUMS-CPS from 1968 forward.<br><br>For general discussion of the IND1950 variable, users should consult the IPUMS-USA documentation. For discussion of the CPS data on industry that are recoded into IND1950, see IND.
Concept:	Work Variables -- PERSON
Start Position:	144
End Position:	146

Width:	3
Variable Format:	numeric
Implied Decimal Places:	0

Categories

Value	Label
000	NIU
105	Agriculture
116	Forestry
126	Fisheries
206	Metal mining
216	Coal mining
226	Crude petroleum and natural gas extraction
236	Nonmetallic mining and quarrying, except fuel
246	Construction
306	Logging
307	Sawmills, planing mills, and millwork
308	Misc wood products
309	Furniture and fixtures
316	Glass and glass products
317	Cement, concrete, gypsum and plaster products
318	Structural clay products
319	Pottery and related products

326	Miscellaneous nonmetallic mineral and stone products
336	Blast furnaces, steel works, and rolling mills
337	Other primary iron and steel industries
338	Primary nonferrous industries
346	Fabricated steel products
347	Fabricated nonferrous metal products
348	Not specified metal industries
356	Agricultural machinery and tractors
357	Office and store machines and devices
358	Miscellaneous machinery
367	Electrical machinery, equipment, and supplies
376	Motor vehicles and motor vehicle equipment
377	Aircraft and parts
378	Ship and boat building and repairing
379	Railroad and miscellaneous transportation equipment
386	Professional equipment and supplies
387	Photographic equipment and supplies
388	Watches, clocks, and clockwork-operated devices
399	Miscellaneous manufacturing industries
406	Meat products
407	Dairy products
408	Canning and preserving fruits, vegetables, and seafoods
409	Grain-mill products
416	Bakery products

417	Confectionery and related products
418	Beverage industries
419	Miscellaneous food preparations and kindred products
426	Not specified food industries
429	Tobacco manufactures
436	Knitting mills
437	Dyeing and finishing textiles, except knit goods
438	Carpets, rugs, and other floor coverings
439	Yarn, thread, and fabric mills
446	Miscellaneous textile mill products
448	Apparel and accessories
449	Miscellaneous fabricated textile products
456	Pulp, paper, and paperboard mills
457	Paperboard containers and boxes
458	Miscellaneous paper and pulp products
459	Printing, publishing, and allied industries
466	Synthetic fibers
467	Drugs and medicines
468	Paints, varnishes, and related products
469	Miscellaneous chemicals and allied products
476	Petroleum refining
477	Miscellaneous petroleum and coal products
478	Rubber products
487	Leather: tanned, curried, and finished

488	Footwear, except rubber
489	Leather products, except footwear
499	Not specified manufacturing industries
506	Railroads and railway express service
516	Street railways and bus lines
526	Trucking service
527	Warehousing and storage
536	Taxicab service
546	Water transportation
556	Air transportation
567	Petroleum and gasoline pipe lines
568	Services incidental to transportation
578	Telephone
579	Telegraph
586	Electric light and power
587	Gas and steam supply systems
588	Electric-gas utilities
596	Water supply
597	Sanitary services
598	Other and not specified utilities
606	Motor vehicles and equipment
607	Drugs, chemicals, and allied products
608	Dry goods apparel
609	Food and related products

616	Electrical goods, hardware, and plumbing equipment
617	Machinery, equipment, and supplies
618	Petroleum products
619	Farm products--raw materials
626	Miscellaneous wholesale trade
627	Not specified wholesale trade
636	Food stores, except dairy products
637	Dairy products stores and milk retailing
646	General merchandise stores
647	Five and ten cent stores
656	Apparel and accessories stores, except shoe
657	Shoe stores
658	Furniture and house furnishing stores
659	Household appliance and radio stores
667	Motor vehicles and accessories retailing
668	Gasoline service stations
669	Drug stores
679	Eating and drinking places
686	Hardware and farm implement stores
687	Lumber and building material retailing
688	Liquor stores
689	Retail florists
696	Jewelry stores
697	Fuel and ice retailing

698	Miscellaneous retail stores
699	Not specified retail trade
716	Banking and credit agencies
726	Security and commodity brokerage and investment companies
736	Insurance
746	Real estate
806	Advertising
807	Accounting, auditing, and bookkeeping services
808	Miscellaneous business services
816	Auto repair services and garages
817	Miscellaneous repair services
826	Private households
836	Hotels and lodging places
846	Laundering, cleaning, and dyeing services
847	Dressmaking shops
848	Shoe repair shops
849	Miscellaneous personal services
856	Radio broadcasting and television
857	Theaters and motion pictures
858	Bowling alleys, and billiard and pool parlors
859	Miscellaneous entertainment and recreation services
868	Medical and other health services, except hospitals
869	Hospitals
879	Legal services

888	Educational services
896	Welfare and religious services
897	Nonprofit membership organizations
898	Engineering and architectural services
899	Miscellaneous professional and related services
906	Postal service
916	Federal public administration
926	State public administration
936	Local public administration
997	Nonclassifiable
998	Industry not reported

Variable: "CLASSWKR"

Name:	CLASSWKR
Label:	Class of worker
Variable Text:	CLASSWKR indicates whether a respondent was self-employed, was an employee in private industry or the public sector, was in the armed forces, or worked without pay in a family business or farm. Workers with multiple sources of employment were classified according to the job in which they worked the most hours. For persons employed at the time of the survey, CLASSWKR relates to the respondent's job during the previous week. Respondents who were not employed during the previous week reported the most recent job. The CLASSWLY variable deals with the longest job held during the previous calendar year.<br><br>In the original CPS public use data, members of the armed forces are coded as N.I.U. (Not in Universe), because they are not part of the civilian labor force.
Concept:	Work Variables -- PERSON
Start Position:	147
End Position:	148

Width:	2
Variable Format:	numeric
Implied Decimal Places:	0

Categories

Value	Label
00	NIU
10	Self-employed
13	Self-employed, not incorporated
14	Self-employed, incorporated
20	Works for wages or salary
21	Wage/salary, private
22	Private, for profit
23	Private, nonprofit
24	Wage/salary, government
25	Federal government employee
26	Armed forces
27	State government employee
28	Local government employee
29	Unpaid family worker
99	Missing/Unknown

Variable: "WKSWORK1"

Name:	WKSWORK1

Label:	Weeks worked last year
Variable Text:	WKSWORK1 reports the number of weeks, in single weeks, that the respondent worked for profit, pay, or as an unpaid family worker during the preceding calendar year. Respondents were prompted to count weeks in which they worked for even a few hours and to include paid vacation and sick leave as work. Information on weeks worked during the preceding year is available in the form of intervals for 1962 forward in the WKSWORK2 variable.
Concept:	Work Variables -- PERSON
Start Position:	149
End Position:	150
Width:	2
Variable Format:	numeric
Implied Decimal Places:	0
Coder Instructions:	WKSWORK1 is a 2-digit numeric value.

Variable: "UHRSWORKLY"

Name:	UHRSWORKLY
Label:	Usual hours worked per week (last yr)
Variable Text:	UHRSWORKLY reports the number of hours per week that respondents usually worked if they worked during the previous calendar year. Individuals were asked this question if: 1) they reported working at a job or business at any time during the previous year or 2) they acknowledged doing "any temporary, part-time, or seasonal work even for a few days" during the previous year.<br><br>See the Hours Worked Variables Notes [URL omitted from DDI.] for an overview of the different actual and usual hours worked variables available.
Concept:	Work Variables -- PERSON
Start Position:	151
End Position:	153
Width:	3

Variable Format:	numeric
Implied Decimal Places:	0
Coder Instructions:	UHRSWORKLY is a 2-digit numeric value.<br><br>99 = 99 hours or more<br>999 = NIU (Not in universe)

Variable: "UHRSWORKT"

Name:	UHRSWORKT
Label:	Hours usually worked per week at all jobs
Variable Text:	UHRSWORKT is the usual number of hours per week the respondent reports being at all jobs, over an unspecified time period.<br><br>See the Hours Worked Variables Notes [URL omitted from DDI.] for an overview of the different actual and usual hours worked variables available.
Concept:	Work Variables -- PERSON
Start Position:	154
End Position:	156
Width:	3
Variable Format:	numeric
Implied Decimal Places:	0

Categories

Value	Label
997	Hours vary
999	NIU

Variable: "UHRSWORK1"

Name:	UHRSWORK1
Label:	Hours usually worked per week at main job
Variable Text:	UHRSWORK1 is the usual number of hours per week the respondent reports being at their main job. There is no time period specified, unlike for UHRSWORKLY, which specifies the reference time period as last year.<br><br>See the Hours Worked Variables Notes [URL omitted from DDI.] for an overview of the different actual and usual hours worked variables available.
Concept:	Work Variables -- PERSON
Start Position:	157
End Position:	159
Width:	3
Variable Format:	numeric
Implied Decimal Places:	0

Categories

Value	Label
000	0 hours
997	Hours vary
999	NIU/Missing

Variable: "AHRSWORKT"

Name:	AHRSWORKT
Label:	Hours worked last week
Variable Text:	AHRSWORKT reports the total number of hours the respondent was at work during the previous week. For employers and the self-employed, this includes all hours spent attending to their operation(s) or enterprise(s). For employees, it is the number of hours they spent at work. For unpaid family workers, it is the number of hours spent doing work directly related to the family business or farm (not including housework).

	See the Hours Worked Variables Notes [URL omitted from DDI.] for an overview of the different actual and usual hours worked variables.
Concept:	Work Variables -- PERSON
Start Position:	160
End Position:	162
Width:	3
Variable Format:	numeric
Implied Decimal Places:	0
Coder Instructions:	AHRSWORKT is a 3-digit numeric variable.<br><br>999 = NIU (Not in universe)

Variable: "HOURWAGE"

Name:	HOURWAGE
Label:	Hourly wage
Variable Text:	HOURWAGE reports how much the respondent earned per hour in the current job, for those workers paid an hourly wage (and coded as "2" in PAIDHOUR). Amounts are expressed as they were reported to the interviewer; users must adjust for inflation using Consumer Price Index [URL omitted from DDI.] adjustment factors. Researchers should use the EARNWT weight with this variable.<br><br>Users should note that HOURWAGE originally had two implied decimal places, but was revised so that the command files provided by IPUMS divide HOURWAGE by 100.<br><br>HOURWAGE is one of the Outgoing Rotation/Earner Study [URL omitted from DDI.] questions.
Concept:	Outgoing Rotation Groups (Earner Study) Variables -- PERSON
Start Position:	163
End Position:	166
Width:	4

Variable Format:	numeric
Implied Decimal Places:	2
Coder Instructions:	99.99 = N.I.U. (Not in Universe).<br>99.97 = Top Code (Wages of $99.99 an hour or more).<br><br>See User Note [URL omitted from DDI.] for these codes

Variable: "PAIDHOUR"

Name:	PAIDHOUR
Label:	Paid by the hour
Variable Text:	PAIDHOUR is a dichotomous variable indicating whether the respondent was paid by the hour for their current job or not. Researchers should use the EARNWT weight with this variable.<br><br>PAIDHOUR is one of the Outgoing Rotation/Earner Study [URL omitted from DDI.] questions.
Concept:	Outgoing Rotation Groups (Earner Study) Variables -- PERSON
Start Position:	167
End Position:	167
Width:	1
Variable Format:	numeric
Implied Decimal Places:	0

Categories

Value	Label
0	NIU
1	No

2	Yes
6	Refused
7	Don't Know

Variable: "UNION"

Name:	UNION
Label:	Union membership
Variable Text:	UNION indicates whether, for the current job, the respondent was: 1) a member of a labor union or employee association similar to a union; 2) not a union member but covered by a union or employee association contract; or 3) neither a union member nor covered by a union contract. Researchers should use the EARNWT weight with this variable.
Concept:	Outgoing Rotation Groups (Earner Study) Variables -- PERSON
Start Position:	168
End Position:	168
Width:	1
Variable Format:	numeric
Implied Decimal Places:	0

Categories

Value	Label
0	NIU
1	No union coverage
2	Member of labor union
3	Covered by union but not a member

Variable: "FIRMSIZE"

Name:	FIRMSIZE
Label:	Number of employees
Variable Text:	FIRMSIZE indicates the total number of persons who worked for the respondent's employer during the preceding calendar year, counting all locations where the employer operated. If the individual was self-employed in a business or farm, the response to FIRMSIZE indicates how many employees worked for the respondent. Responses were grouped into broad categories, such as "under 25 employees," "25 to 99 employees," "100 to 499 employees," "500 to 999 employees," and "1000+ employees."
Concept:	Work Variables -- PERSON
Start Position:	169
End Position:	169
Width:	1
Variable Format:	numeric
Implied Decimal Places:	0

Categories

Value	Label
0	NIU
1	Under 10
2	10 to 24
3	Under 25
4	10 to 49
5	25 to 99
6	50 to 99

7	100 to 499
8	500 to 999
9	1000+

Variable: "INCWAGE"

Name:	INCWAGE
Label:	Wage and salary income
Variable Text:	INCWAGE indicates each respondent's total pre-tax wage and salary income--that is, money received as an employee--for the previous calendar year. Amounts are expressed as they were reported to the interviewer; users must adjust for inflation using Consumer Price Index [URL omitted from DDI.] adjustment factors.<br><br>For ASEC samples 1988-onward, INCWAGE is derived from a Census recode variable. The topcoded components of INCWAGE are OINCWAGE and INCLONGJ. OINCWAGE is always a component of INCWAGE. When SRCEARN indicates that INCLONGJ is earned from wage and salary, INCLONGJ is an additional component of INCWAGE.
Concept:	Income Variables -- PERSON
Start Position:	170
End Position:	176
Width:	7
Variable Format:	numeric
Implied Decimal Places:	0
Coder Instructions:	9999999 = N.I.U. (Not in Universe).<br>9999998 = Missing.<br><br>IPUMS does not identify topcodes in the data. Refer to the Topcodes Tables link below for income topcodes documentation.<br><br>See User Note [URL omitted from DDI.] for an explanation of these codes, and the Topcodes Tables [URL omitted from DDI.] page for documentation on income component topcodes over time.

Variable: "INCFARM"

Name:	INCFARM
Label:	Farm income
Variable Text:	INCFARM indicates each respondent's net pre-income-tax earnings as a tenant farmer, sharecropper, or operator of his or her own farm during the previous calendar year. INCFARM collects income information for self-employed persons who had their own farms. Income earned as an employee on a farm is contained in the variable INCWAGE.<br><br>Amounts are expressed as they were reported to the interviewer; users must adjust for inflation using Consumer Price Index [URL omitted from DDI.] adjustment factors.<br><br>For ASEC samples 1988-onward, INCFARM is derived from a Census recode variable. The topcoded components of INCFARM are OINCFARM and INCLONGJ. OINCFARM is always a component of INCFARM. When SRCEARN indicates that INCLONGJ is earned from farm self-employment, INCLONGJ is an additional component of INCFARM.
Concept:	Income Variables -- PERSON
Start Position:	177
End Position:	183
Width:	7
Variable Format:	numeric
Implied Decimal Places:	0
Coder Instructions:	9999999 = N.I.U. (Not in Universe).<br>9999998 = Missing.<br>Values can be negative.<br><br>IPUMS does not identify topcodes in the data. Refer to the Topcodes Tables link below for income topcodes documentation.<br><br>See User Note [URL omitted from DDI.] for an explanation of these codes, and the Topcodes Tables [URL omitted from DDI.] page for documentation on income component topcodes over time.

Variable: "EARNWEEK"

Name:	EARNWEEK
Label:	Weekly earnings

Variable Text:	EARNWEEK reports how much the respondent usually earned per week at their current job, before deductions. Interviewers asked directly about total weekly earnings and also collected information about the usual number of hours worked per week and the hourly rate of pay at the current job. The figure given in EARNWEEK is the higher of the values derived from these two sources: 1) the respondent's answer to the question, "How much do you usually earn per week at this job before deductions?"; or 2) for workers paid by the hour (and coded as "2" in PAIDHOUR), the reported number of hours the respondent usually worked at the job, multiplied by the hourly wage rate given in HOURWAGE.<br><br>The values in EARNWEEK are in dollars, with no implied decimal places; a value of 500 means that the respondent earned five hundred dollars per week before deductions. Amounts are expressed as they were reported to the interviewer; users must adjust for inflation using Consumer Price Index [URL omitted from DDI.] adjustment factors. Researchers should use the EARNWT weight with this variable.<br><br>EARNWEEK is one of the Outgoing Rotation/Earner Study [URL omitted from DDI.] questions.
Concept:	Outgoing Rotation Groups (Earner Study) Variables -- PERSON
Start Position:	184
End Position:	191
Width:	8
Variable Format:	numeric
Implied Decimal Places:	2
Coder Instructions:	9999.99 = N.I.U. (Not in Universe).<br><br>1990-1997: 1923 (Weekly earnings of $1923 or more).<br>1998-onward: 2885 (Weekly earnings of $2885 or more: ASEC samples only).<br>2884.61 for non-ASEC samples.<br><br>See User Note [URL omitted from DDI.] for an explanation of these codes.

Variable: "OINCWAGE"

Name:	OINCWAGE
Label:	Earnings from other work included wage and salary earnings
Variable Text:	OINCWAGE reports the net amount (prior to deductions) that the respondent earned in wages and salary, other than the amount earned in the primary (longest-held) job, during the preceding calendar year. The amount earned (in wages, salary, or earnings

	from self-employment in a business or farm) from the longest held job during the preceding calendar year is reported in INCLONGJ.<br><br>Imagine that an individual worked at one job for 8 months, earning $10,000 in wages, and worked at two other jobs for the remaining 4 months, earning $12,000 in wages. The $12,000 in "other" wage and salary earnings (wages and salary from jobs other than the person's primary, or longest-held, job) would be reported in OINCWAGE; the $10,000 in wages from the individual's longest-held job would be reported in INCLONGJ; and the total amount of wage and salary income from the previous calendar year ($22,000) would be reported in INCWAGE.<br><br>Imagine, instead, that as reported in SRCEARN, an individual was self-employed in a business or farm for their primary job, and also worked one or more additional jobs for wages. In such a case, self-employment income from the primary job would be reported in INCLONGJ; all wage and salary earnings would be reported in OINCWAGE, and the value of OINCWAGE and INCWAGE would be the same.<br><br>Amounts are expressed as they were reported to the interviewer; users must adjust for inflation using Consumer Price Index [URL omitted from DDI.] adjustment factors.
Concept:	Income Variables -- PERSON
Start Position:	192
End Position:	198
Width:	7
Variable Format:	numeric
Implied Decimal Places:	0
Coder Instructions:	9999999 = N.I.U. (Not in Universe).<br><br>99997 = Topcode (1988-1995). For other years, see the Topcodes Tables link below.<br><br>See User Note [URL omitted from DDI.] for an explanation of these codes, and the Topcodes Tables [URL omitted from DDI.] page for documentation on income component topcodes over time.

Variable: "WKSTAT"

Name:	WKSTAT
Label:	Full or part time status
Variable Text:	WKSTAT is a recode from the Census Bureau that states the part-time or full-time employment status for the respondent, and reasons. It is derived from a number of

	labor force questions asked in the monthly questionnaire.
Concept:	Work Variables -- PERSON
Start Position:	199
End Position:	200
Width:	2
Variable Format:	numeric
Implied Decimal Places:	0

Categories

Value	Label
10	Full-time schedules
11	Full-time hours (35+), usually full-time
12	Part-time for non-economic reasons, usually full-time
13	Not at work, usually full-time
14	Full-time hours, usually part-time for economic reasons
15	Full-time hours, usually part-time for non-economic reasons
20	Part-time for economic reasons
21	Part-time for economic reasons, usually full-time
22	Part-time hours, usually part-time for economic reasons
40	Part-time for non-economic reasons, usually part-time
41	Part-time hours, usually part-time for non-economic reasons
42	Not at work, usually part-time
50	Unemployed, seeking full-time work

60	Unemployed, seeking part-time work
99	NIU, blank, or not in labor force
